# Unveiling Coformulants in Plant Protection Products by LC-HRMS Using a Polyhydroxy Methacrylate Stationary Phase

**DOI:** 10.1021/acs.jafc.3c03600

**Published:** 2023-10-17

**Authors:** Beatriz Martín-García, Roberto Romero-González, José Luis Martínez Vidal, Antonia Garrido Frenich

**Affiliations:** Research group “Analytical Chemistry of Contaminants”, Department of Chemistry and Physics, Research Centre for Mediterranean Intensive Agrosystems and Agri-Food Biotechnology (CIAMBITAL), University of Almería, Agri-Food Campus of International Excellence, ceiA3, 04120 Almería, Spain

**Keywords:** plant protection products, coformulants, HPLC-Q-Orbitrap-MS/MS, suspect screening, unknown analysis

## Abstract

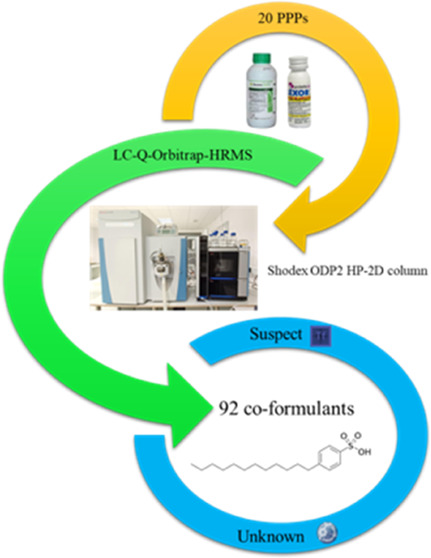

A polyhydroxy methacrylate-based stationary reversed phase was used for the determination of coformulants in 20 plant protection products (PPPs). These samples were analyzed by liquid chromatography coupled to Q-Orbitrap high-resolution mass spectrometry (LC-Q-Orbitrap-HRMS) in full-scan MS and data-dependent acquisition (ddMS^2^) modes. A total of 92 coformulants were tentatively identified in these formulations by nontargeted and unknown analyses. Twelve out of them were quantified by analytical standards. The most concentrated coformulant was the anionic surfactant dodecylbenzenesulfonic acid, whose highest content was obtained in the Score 25 sample (6.87%, w/v). Furthermore, triethylene glycol monomethyl ether, 4-*s*-butyl-2,6-di-*tert*-butylphenol, 1-ethyl-2-pyrrolidone, sorbitan monostearate, 2,6-dimethylaniline, palmitamide, and N-lauryldiethanolamine were quantified for the first time in these products. Hence, the polyhydroxy methacrylate-based stationary phase increased the identification of new coformulants in PPPs, being complementary to conventional C18. This strategy could be applied in future studies to estimate potential coformulant residues from PPPs applied to crops.

## 1 Introduction

Plant protection products (PPPs) have long been essential resources for effective pest control. According to recent month pesticide sales from EUROSTAT data, up to 393 million tons (Mt) of the PPPs were sold in the EU-27 in 2021.^1^ Spain leads the sale of pesticides in the European Union (EU), with about 76 Mt in 2020, ahead of France, Italy, and Germany, and these four countries are the main agricultural producers in the EU, representing 68% of the total sales. PPPs are mainly composed of active substances but they also contain a wide variety of coformulants.^2^ These give PPPs the qualities they need for application, thereby improving the effectiveness of the active ingredients. As far as the toxicity of PPPs, active substances are often considered to be the main cause of toxicity. Thus, regulation (EU) 283/2013 only requires extensive mammalian toxicity testing for acute, chronic, and subchronic effects of the active substances.^3^ As part of PPP Regulation (EU) 1107/2009, the coformulants employed therein do not need any further specific toxicological evaluation or authorization.^4^ Nevertheless, recent studies have shown that PPPs possess a higher toxicity in comparison with their active substances.^5−7^ This fact is due to the interaction between the active substance and safeners, synergists, and coformulants that may increase the toxicity of PPP. For that reason, Commission Regulation (EU) 2021/383 March 3 modified Annex III to Regulation (EC) No 1107/2009 of the European Parliament and of the Council, where a list of 144 coformulants that cannot be included in the composition of the phytosanitary products is published.^8^ Around 60% of these banned substances in PPP correspond with nonyl-phenols and octyl-phenols and their ethoxylated derivatives that possess endocrine-disrupting properties. In addition, dibutyl phthalate has endocrine-disruption properties and it may have harmful reproductive effects. Other abundant coformulants, including solvents such as naphtha, lubricant oils, and distillates derived from petroleum distillation, have shown carcinogenic effects.^8^ Apart from those listed in Annex III of Regulation (EC) no 1107/2009, there are coformulants that are not authorized in Spain to be used in PPP. Among them, there are substances such as 4-methylpentan-2-one, isobutyl methyl ketone, aniline, isophorone, naphthalene, and tributyl phosphate that possess carcinogenic effects at concentrations equal to or higher than 1%.^9^

In addition, certain coformulants can contain impurities with toxicological relevance or components of concern, such as benzene, 1,3-butadiene, polycyclic aromatics, benzo(a)-pyrene, crystalline silica, or asbestiform fibers. Therefore, concentrations of these coformulants should be below 0.1% (*w/w*) or below-specified concentration limits for carcinogenicity, mutagenicity, and reproductive toxicity.^8^

There are recent studies that have used HRMS to carry out nontargeted or suspect analyses as a powerful tool to determine a wide range of coformulants present in PPPs. Maldonado-Reina et al. tentatively identified 42 compounds by gas chromatography (GC) coupled to Q-Orbitrap-HRMS by suspect screening and unknown analysis in 14 PPPs corresponding to several types of formulations.^10^ Another study analyzed six commercial pesticide formulations with antifungal activity by GC-Q-Orbitrap that allowed the quantification of 21 compounds.^11^ López-Ruiz et al. employed LC and GC coupled to an Exactive Orbitrap-MS analyzer to determine nine adjuvants in three emulsifiable concentrates (ECs) applying a suspect screening approach.^12^ Among them, nonaethylene glycol monododecyl ether, sodium dodecyl sulfate, and glyceryl monostearate were characterized by LC-HRMS.^12^ Balmer et al. selected four common coformulants in three different PPPs to quantify their residues in vegetables and apples under field conditions using LC-MS/MS.^13^ In addition, other studies have used LC-Q-Orbitrap-MS to determine the presence of coformulants in different PPPs.^2,14^ Hergueta-Castillo et al. determined six coformulants,^14^ whereas Maldonado-Reina et al. tentatively identified 78 coformulants, and nine of them were confirmed by analytical standards.^2^ These previous studies used a C18 column,^2,14^ which has been shown to be effective for the separation of nonionic surfactants including alkyl ethoxylates, isothiazolone (1,2-benzisothiazol-3(2H)-one), and other hydrophobic compounds, such as glyceryl monosterate among others. However, the C18 stationary phase does not offer the best selectivity to analyze anionic, nonionic, and cationic surfactants simultaneously with the same mobile phase. The separation of these substances may be improved by using additional stationary phases specifically developed for the separation of surfactants. A previous study used the Acclaim Surfactant Plus column method for the determination of an anionic, cationic, and amphoteric surfactant mixture from surface water samples by LC coupled with charged aerosol detection (CAD).^15^ Another study determined anionic, cationic, and nonionic surfactants in surface water by LC-MS using two methods, utilizing Acclaim Surfactant Plus and Poroshell 120 EC-C18 column.^16^ This study reported a difference in the chromatographic peaks in which the C18 column presented sharper peaks with a more Gaussian shape than the Acclaim column.^16^ Furthermore, Shodex ODP2 HP series columns were utilized for the analysis of aggregates in antibody drugs by LC-MS, including nonionic surfactants such as polysorbate 20 and 80.^17,18^ The Shodex ODP2 HP columns have an efficiency comparable to that of silica-based octadecyl columns and are more efficient than the majority of resin-based columns. These types of columns do not contain C18 functional groups; the separation comes from the particle itself. Compared to the majority of general-purpose silica-based C18 columns, Shodex ODP2 HP offers superior retention of highly polar compounds and enhanced retention of highly polar substances compared with the retention of the high polar substances compared to the ODS columns. Therefore, this type of column is suitable for LC/MS analysis of high polar compounds.^19^ Nevertheless, to the best of our knowledge, there are no studies that have used this type of column to analyze additives, such as coformulants in PPPs. Therefore, the objective of this study was the determination of coformulants in 20 PPPs by using a new method based on the use of a Shodex ODP2 HP-2D as a stationary phase that offers a good separation of hydrophilic substances by LC-HRMS, although an Acclaim Surfactant Plus column was also tested. The results were compared with previous results obtained by using a Hypersil GOLD aQ column as a stationary phase to analyze coformulants in the same PPPs.^2,14^

## 2 Materials and Methods

### 2.1 Equipment, Materials. and Reagents

Table S1 shows the composition of the active ingredients in the 20 PPPs. The formulation types are emulsifiable concentrate (EC), emulsion in water (EW), suspension concentrate (SC), water-dispersible granule (WG), dispersible concentrate (DC), and a mixture of capsule suspension (CS) in SC (ZC). These PPPs were as follows: P1: Voliam Targo (SC), P2: Kabuto JED (EC), P3: Mavita 250 (EC), P4: Cidely Top (DC), P5: Dynali (DC), P6: Lexor 25 (EC), P7: Score 25 EC (EC), P8: Dagonis (SC), P9: Coragen 20 SC (SC), P10: Altacor 35(WG), P11: Ampligo 150 (ZC), P12: Nomada (EC), P13: Duaxo (EC), P14: Ortiva Top (SC), P15: Flint Max (WG), P16: Topas (EW), P17: Massocur 12.5 (EC), P18: Impact Evo (SC), P19: Latino (MITRUS, EC), and P20: Impala Star (EW).

Regarding analytical grade standards, sodium dodecyl benzenesulfonate (CRM, 100%) and aniline (≥99.5%) were supplied by Sigma-Aldrich (St. Louis, MO). 4-*sec*-Butyl-2,6-di-*tert*-butylphenol (>98.0%), triethylene glycol monomethyl ether (>98%), 1-ethyl-2-pyrrolidone, and Span-60 (sorbitan monostearate) were acquired from TCI (Zwijndrecht, Belgium). Naphthalene-1-sulfonic acid sodium salt and 2,6-dimethylaniline (99%) were supplied by Alfa Aesar (Ward Hill, MA), whereas lauramide DEA (≥95.0%) and palmitamide (>95%) were purchased from Fluorochem (Hadfield, United Kingdom). Xylene (99.3%) was supplied by Dr. Ehrenstorfer (Augsburg, Germany), and methyl 3-(3,5-di-*tert*-butyl-4-hydroxyphenyl)propionate (Metilox) (98%) by Tokyo Chemical Industry (Nihonbashi-Honcho, Chuo-Ku, Tokyo, Japan).

Methanol (LC-MS Chromasolv, ≥99.9%), purchased from Honeywell (Charlotte, NC), water (LC-MS LiChromasolv), obtained from Merck (Darmstadt, Germany), and acetonitrile (LC-MS Chromasolv, ≥99.9%), supplied by Honeywell, were used to dissolve the PPPs or to prepare the mobile phase. Ammonium acetate and ammonium hydroxide (LC-MS, 99.0%) were acquired from Fischer Scientific (Waltham, MD). The internal standard caffeine-^13^C_3_ was purchased from Supelco Sigma-Aldrich (St. Louis, MO). Caffeine-^13^C_3_ is a stable isotope internal standard commonly used in LC-MS as an internal standard. Additionally, it is a polar compound with a low value of LogP, which is similar to some of the compounds detected in this study.

The LC equipment employed was a Thermo Fisher Scientific Vanquish Flex Quaternary LC (Thermo Fisher Scientific) coupled to a Q-Exactive Orbitrap (Thermo Fisher Scientific) mass spectrometer. The mass calibration of the Q-Orbitrap analyzer was carried out by using a mixture of acetic acid, caffeine, Met–Arg–Phe–Ala–acetate salt, and Ultramark 1621 (ProteoMass LTQ/FT-hybrid ESI positive and negative) from Thermo Fisher (Waltham, MA).

### 2.2 Sample Processing

The dilution of PPPs was carried out according to Maldonado-Reina et al.^2^ Briefly, 40 μL aliquots of each PPP were diluted in 4 mL of water (dilution of 100 v/v), and this solution was shaken for 1 min in a vortex mixer. Then, 100 μL of this solution was diluted in 900 μL of a 50:50 methanol/water mixture (v/v) to obtain a dilution of 1000 (v/v). This last solution was diluted at 1:10 (v/v) to obtain a final dilution of 10,000 (v/v). For this purpose, 100 μL of the dilution 1000 (v/v) was diluted in 900 μL of the mixture (850 μL of methanol/water 50:50 and 50 μL of the internal standard (caffeine) at 1 mg/L in methanol). The final concentration of caffeine was 50 μg/L. The final dilution of 10,000 (v/v) was filtered with nylon syringe filters (0.20 μm pore size) and injected into the LC system. Altacor 35 and Flint Max were solid PPPs in the form of granules (WG formulation), and 40 mg of these products was weighed and dissolved in 4 mL of water. These solutions were prepared in the same way as the previous liquid PPPs to achieve a final dilution of 10,000 (w/v).

### 2.3 LC-Q-Orbitrap-MS and LC-Q-Orbitrap-MS^2^ Conditions

Two columns were first tested to compare the separation of coformulants in the PPPs: Shodex ODP2 HP-2D (2 mm × 150 mm, 5 μm) (Symta, Madrid, Spain) composed of a polyhydroxy methacrylate and Acclaim Surfactant Plus was a silica-based mixed mode column (2.1 mm × 100 mm, 3 μm) (Thermo Fisher Scientific, Waltham, MA).

In the case of Shodex ODP2 HP-2D, the mobile phase was an aqueous solution of ammonium hydroxide (0.1%) as component A and acetonitrile as component B. The flow rate was 0.2 mL/min and the injection volume was 10 μL. The gradient conditions were the following: 20% B from 0 to 5 min, increased up to 90% B from 5 to 19 min, and remained constant for 5 min, decreasing to 20% B during 1 min. The equilibration time was 2.0 min after returning to the initial conditions; therefore, the total run time was 27.0 min.

The gradient conditions for the Acclaim Surfactant Plus were established according to the method for a simultaneous analysis of cationic, nonionic, amphoteric, and anionic surfactants by LC-ESI-MS reported by the Thermo Scientific Acclaim Surfactant Plus Product Manual.^20^ The mobile phase was water (phase A), 100 mM ammonium acetate at pH 5 (phase B), and acetonitrile (phase C). The flow rate was 0.3 mL/min and the injection volume was 10 μL. The gradient conditions are given in Table S2.

The detection was carried out using an HRMS analyzer (Q-Exactive Orbitrap, Thermo Fisher Scientific, Bremen, Germany) with an electrospray interface (ESI; HESI-II, Thermo Fisher Scientific) in positive and negative ionization modes. ESI conditions were: a capillary temperature of 300 °C, a heater temperature of 305 °C, sheath gas (N_2_, 95%), 35 arbitrary units; auxiliary gas (N_2_, 95%), 10 arbitrary units, a spray voltage of 4 kV, S-lens radio frequency (RF) level, 50 arbitrary units. Full-Scan MS data were acquired in the *m*/*z* range from 90 to 1300, at a resolution of 70,000 at *m*/*z* 200 and an AGC target of 10^6^; ddMS^2^ was performed with a resolution of 35,000 at *m*/*z* 200 and an AGC target value of 10^5^, a loop count of 5, and an isolation window of *m*/*z* 5.0. Software Xcalibur Sequence Setup was used to collect all of the data.

### 2.4 Data Treatment

Xcalibur version 3.0 was used to process the chromatograms employing Quan Browser and Qual Browser. Mass Frontier 8.0 (Thermo Fisher Scientific, Les Ulis, France) was used for in silico fragmentation. TraceFinder version 4.0 (Thermo Fisher Scientific) was employed for suspect screening analysis. Compound Discoverer 3.2 (Thermo Fisher Scientific) was used for unknown analysis utilizing different ChemSpider libraries (EPA DSST and FDA- UNIII-NLM). The validation of the analytical method was carried out according to the parameters described by the document SANTE/11312/2021 to the analytical quality control and method validation for pesticide residue analysis in food and feed.^21^ The liquid samples or solid samples that were completely dissolved were analyzed, and only a dilution step was applied before LC analysis. Therefore, recovery, precision, limit of quantification (LOQ), and matrix effect (ME) were evaluated to validate the method. Intraday precision (%) was evaluated by analyzing five mixtures of standards at 50 μg/L, whereas the interday precision was performed by the injection of the standard mixture at 50 μg/L for 5 consecutive days.

The recovery was evaluated by spiking an aliquot of Kabuto JED with a mixture of standard compounds in order to obtain a final concentration of 50 μg/L. The quantity of each compound was evaluated by subtracting the one that was not spiked and comparing it with the one that was prepared with the solvent at 50 μg/L if the matrix effect was negligible. If not, then standard addition methodology, as indicated below, was applied.

The linearity of the confirmed coformulants in PPPs was carried out by the calibration curves of each standard, and these were prepared in methanol in a concentration range from LOQ to 100 μg/L. A dilution of each standard solution from the stock solution at 1000 to 10 mg/L was carried out. Following that, 100 μL of each standard solution at 10 mg/L was diluted in a final volume of 10 mL to produce a final combination solution with a concentration of 100 μg/L for each standard. Based on this, several concentrations were used for the calibration curves, from the LOQ to 100 μg/L obtained from the 100 μg/L mixture. Caffeine-^13^C_3_ was used as the internal standard at 50 μg/L and the calibration curves were carried out by using the peak area analyte standard/area of an internal standard against the concentration of each standard, except for those compounds ionized in negative mode. In order to evaluate the ME, the samples of PPPs were fortified with 50 μg/L of a mixture of the analytical standards. Matrix effects were obtained by subtracting the area of the sample fortified with the area of the sample (unfortified), and then comparing it with its standard solvent. The matrix effect was calculated according to the following formula (eq 1)
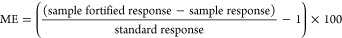
1In case the matrix effect was significant, standard addition methodology was used to estimate the concentration of the identified compounds in each PPP, injecting the diluted sample and adding the same concentration levels as those used in the estimation of the linearity.

#### 2.4.1 Suspect Screening

Full-scan MS was selected to acquire the total ion chromatogram (TIC) of the compounds. In addition, fragment ions were obtained by data-dependent acquisition (ddMS^2^). Data obtained from LC-Q-Orbitrap were processed with TraceFinder software, which enables retrospective analysis using an extensive coformulant homemade database of 264 compounds obtained from previous studies and Regulation (EU) 284/2013^2,14,22^ (Table S3). An extensive range of coformulants, including solvents, alkyl ethoxylates, preservatives, anionic and nonionic surfactants, alcohols or nonylphenol and octylphenol derivatives, and other types of coformulants, were included in this database. Then, these suspect compounds were carefully searched in all PPPs using either their molecular ions ([M + H]^+^ or [M – H]^−^) or characteristic adduct ions (such as [M + NH_4_]^+^). During the identification process, the Schymanski criteria based on confidence by different levels was applied.^23^

#### 2.4.2 Unknown Analysis

An unknown analysis was carried out to identify compounds not included in the previous database, as well as to check coformulants that have been detected by the suspect screening strategy. Therefore, an unknown analysis was carried out by Compound Discoverer, using ChemSpider libraries (Alfa Chemistry, Alkamid, Aurora Fine Chemicals, Environmental Protection Agency Distributed Structure-Searchable Toxicity (EPA DSSTox), Chemspace, EPA Toxcast, FDA, Food and Drug Administration Unique Ingredient Identifier from the National Library of Medicine (FDA UNII-NLM), FooDB, KEGG, MassBank, Molbank, Nature Chemical Biology, and Nature Chemistry) and mzCloud. The identification of these compounds was achieved by taking into account a mass accuracy limit of 10 ppm and an appropriate peak shape signal. In the absence of noise, a signal must be present in at least five subsequent scans per peak of each ion with a mass error not exceeding 10 ppm. The retention time of fragment ions was equal to the corresponding precursor ion, and the mass error was lower than 10 ppm.

## 3 Results and Discussion

### 3.1 Strategies for Data Processing

#### 3.1.1 Selection of a Stationary Phase

A comparison between two LC methods that involved two types of columns, Shodex ODP2 HP-2D and Aclaim Surfactant Plus, was carried out. For that purpose, the identification of coformulants in Lexor and Score 25 was performed using both stationary phases through the suspect screening and unknown analysis. The method employing Shodex ODP2 HP-2D allowed the tentative identification of 45 coformulants in Lexor and 50 compounds in Score 25 by using suspect and unknown strategies. On the contrary, the method that used the column Acclaim Surfactant Plus only allowed the identification of 6 compounds in Lexor and 5 in Score 25 by both strategies. These coformulants were anionic surfactants, octyl 4-methylbenzenesulfonate, and ethoxylated alcohols including 2-[2-(4-octylphenoxy)ethoxy]-ethanol and other compounds (*N*,*N*-dimethyldecanamide, citric acid, dibutyl phthalate, lauramide DEA and diethylene glycol *n*-butyl ether). These compounds were also identified with a Shodex column. Table S4 shows the identification parameters of coformulants detected by Aclaim Surfactant Plus. This column showed higher retention time for the same compounds identified with Shodex (Table S5), except for the polar compounds 2-[2-(4-octylphenoxy)ethoxy]-ethanol, citric acid, and diethylene glycol *n*-butyl ether, whose retention times were 1.33, 0.67, and 1.62 min respectively, which were lower than those obtained by the Shodex column. This could be explained because the Shodex column is more suitable for polar compounds because this type of column has the capacity to retain compounds with high polarity. In addition, the method employed with the Acclaim column showed lower sensitivity than Shodex, as can be observed in Figure S1, where the extracted ion chromatogram of octyl 4-methylbenzenesulfonate with Shodex (A) and Acclaim column (B) and their corresponding spectra (C-E) are shown. It could be observed that this compound elutes at higher retention times with worse resolution and lower sensitivity in comparison with Shodex. Furthermore, the method with the Shodex column also allowed the detection of other coformulants that were abundant in most of the tested PPPs. These compounds corresponded with anionic surfactants, including dodecylbenzenesulfonic acid and 2-naphthalenesulfonic acid, and alkyl ethylene glycol esters such as triethylene glycol monometyl ether and alkyl amines (*N*-lauryldiethanolamine). However, these coformulants were not detected with Acclaim surfactant Plus. Furthermore, the analysis time with Shodex was 27.0 min, which was shorter than that with Acclaim. As a result, the method that used the Shodex column has been selected since it was able to detect a greater number of compounds in a shorter time than the method developed with the Acclaim column. In the next sections, the results obtained from the identification of coformulants with the Shodex column using the two data processing strategies of suspect screening and unknown are described.

#### 3.1.2 Suspect Screening

A total of 70 coformulants were tentatively detected by the suspect screening (Table S5). According to the Schymanski criteria level of confidence,^23^ 42 compounds belong to level 2, 5 compounds to the level 3, whereas 23 compounds were identified at level 4.

Among the total 70 tentative compounds, 26 were identified for the first time in these PPPs. These coformulants included anionic surfactants with a sulfate group as laureth-2 sulfate, sodium xylenesulfonate, and diisopropylnaphthalenesulfonic acid (compounds 18, 62 and 67), a nonionic surfactant such as alkylphenolethoxylates (compounds 42 and 48), phenoxyethanols (2-(p-octylphenoxy)ethanol, phenoxyethanol, nonylphenoxyethanol), sorbitan monostearate and ethylene glycol (compounds 29 and 31), and amphoteric surfactant such as cocamide propyl betaine. Other coformulants, such as alkyl and phenyl amines (triethanolamine and 2,6-dimethylaniline), alkyl aldehydes and derivatives (3-hexenal, 2-phenylpropanal, 2,2-dimethylocta-3,4-dienal), alkyl alcohol (3,6,9,12-tetraoxapentacosan-1-ol), 2-methylisothiazolone, 1-ethyl-2-pyrrolidone, glutaric anhydride, citric acid, quinoline, butanedioic acid [(3.5-dimethoxyphenyl)methylene]-1-methyl ester, cocamide monoethanolamide xylene, and dibutyl phthalate were also detected. The criteria to select the fragment ions were the most abundant ions and were confirmed with the fragments obtained by Mass Frontier, retention time, which must be equal to the corresponding precursor ion with a retention time shift of ±0.1 min and a mass error of lower than 10 ppm. Table S5 shows the typical parameters found for the suspect compounds.

Regarding the fragmentation of the characteristic ions, a common fragment at *m*/*z* 79.95736 was found for the anionic surfactants including dodecylbenzenesulfonic acid, 4-octylbenzenesulfonic acid, naphthalenesulfonic acid, 2,6-di-*tert*-butylnaphthalene-1-sulfonic acid and laurteh-2-sulfate, which corresponds with a radical sulfate anion (SO_3_^•–^).^24^ Furthermore, the fragment ions of coformulants that were identified for the first time in these products were included when they were detected. For instance, laureth-2 sulfate at *m*/*z* 353.2003 [M – H]^−^ possessed a fragment ion at *m*/*z* 97.06589 (C_6_H_9_O^–^), which corresponded with 5-hexenal. Dibutyl phthalate at *m*/*z* 279.1591, [M + H]^+^, has shown the most abundant fragment ion at *m*/*z* 149.0233 (C_8_H_5_O_3_^+^). Sorbitan monostearate has been identified in positive and negative modes in PPPs. In the case of sorbitan monostearate [M – H]^−^, the most abundant fragment ion was obtained at *m*/*z* 279.23295, which was derived from the loss of the sorbitan group, obtaining octadecadienoic acid (C_18_H_32_O_2_^–^). 1-Ethyl-2-pyrrolidone was detected at 2.1 min with an *m*/*z* of 114.0913 [M + H]^+^, which had a fragment ion at *m*/*z* 112.07569 that corresponded with the loss of a hydrogen (C_6_H_10_NO^+^). Cocamide betaine at *m*/*z* 343.2955 [M + H]^+^ showed an abundant fragment ion at *m*/*z* 240.231537 (C_15_H_30_NO^+^) (dodecanoylamino propyl) and a less abundant fragment ion at *m*/*z* 183.173828 (C_12_H_23_O^+^) (dodecanoylamino). 2-Amino-1,3-dimethylbenzene at *m*/*z* 122.0964 [M + H]^+^ possessed two fragment ions at *m*/*z* 105.06992 (C_8_H_9_^+^) and 107.0731 (C_6_H_7_N_2_^+^), being the most abundant fragment obtained at *m*/*z* 105.06992 (2,3-dimethylbenzene), derived from the loss of the amine group. Citric acid at *m*/*z* 191.0197 [M – H]^−^ was detected in Flint Max, and fragments ions were *m*/*z* 129.0193, 111.0088, and 87.0088, the fragment ion at *m*/*z* 111.0088 being the most abundant.

Fragments of other coformulants that were detected previously in the PPPs^2,14^ have been included; for example, *N*,*N*-dimethyldecanamide was detected at *m*/*z* 200.2009 [M + H]^+^ in five PPPs (Cidely Top, Dynali, Topas, Massocur 12.5 EC and Impala Star). This molecule had the most abundant fragment ion at *m*/*z* 102.09134 (C_5_H_12_ON^+^), which matches with (dimethylamino)acetone derived from the breakage of the carbon C3 linkage. Another abundant fragment ion was detected at *m*/*z* 198.18524, which is obtained by the protonation in *N*,*N*-dimethyldecanamide at the C3 position. In addition, lauramide DEA (*N*,*N*-bis(2-hydroxyethyl)dodecanamide) was detected in nine PPPs (Voliam Targo, Kabuto JED, Dynali, Coragen 20 SC, Altacor, Ampligo, Duaxo, Massocur 12.5 EC, Impala Star) at *m*/*z* 288.25332 [M + H]^+^. This molecule possessed the most abundant fragment ion at *m*/*z* 106.08626 (C_4_H_12_O_2_N^+^) that corresponded to *N*,*N*-bis(2-hydroxyethyl)amine obtained from the breakage of amide C–N bonds. This compound was detected previously only in Altacor.^2^

**Figure 1:**
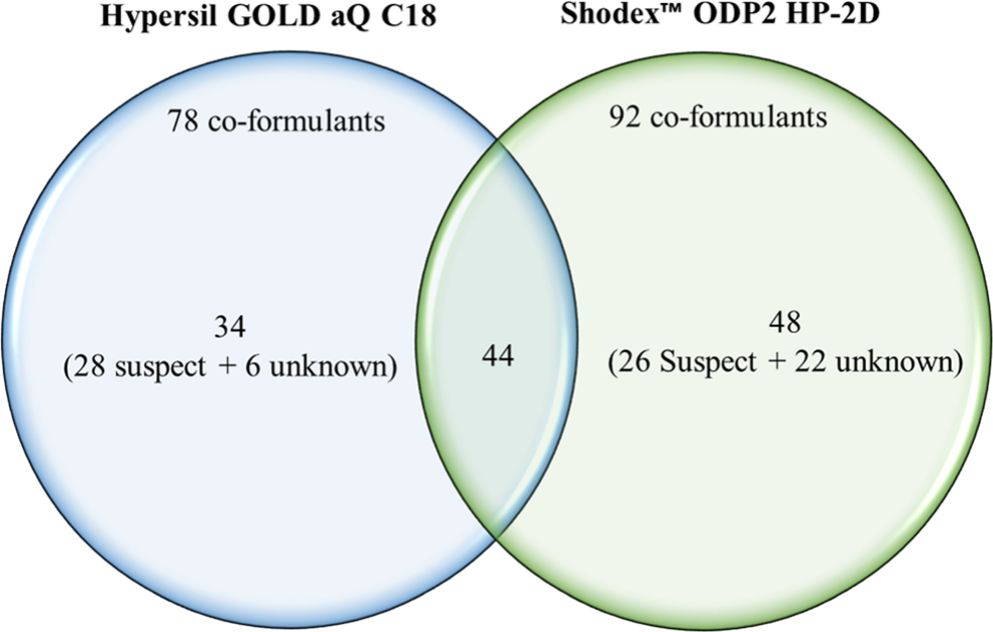
Venn diagram for the comparison of coformulants detected using Shodex ODP2 HP-2D with those detected previously by Hypersil GOLD aQ C18.

It is important to mention that previous studies that used the C18 column as a stationary phase identified 34 compounds that were not identified in the present study^2,14^ (Figure 1). Six of them were confirmed by means of using standards including 1,2-benzisothiazol-3(2*H*)-one, 1-dodecyl naphthalene, myreth-6, monopalmitin, glyceryl monosterate, and dimethyl sulfoxide. The remaining 28 compounds were tentatively identified in these PPPs. These tentative compounds were mainly nonionic coformulants such as poly(ethylene oxide) and derivatives, alkyl ethylene glycol ethers, alkyl naphthalene such as hexadecyl naphthalene, thiazoles such as methylchloroisothiazolinone, alkyl glycol ethers (dipropylene glycol methyl ether), alkyl phenoxy alcohols, and other compounds. Therefore, these previous results showed that column-type C18 is better suited for the separation of other nonionic surfactants, such as thiazoles, poly(ethylene oxide) with long chains, alkyl naphthalene, and other certain hydrophobic compounds such as monopalmitin and glyceryl monosterate, which were not identified with Shodex ODP2 HP-2D. This fact could be related to the hydrophilic nature of the stationary phase in the Shodex ODP2 HP-2D column. Consequently, the use of the two columns could provide a full characterization of coformulants in PPPs; hence, the methodology with Shodex may complement the use of conventional C18 stationary phases.

#### 3.1.3 Unknown Analysis

A total of 396 compounds were detected by unknown analysis mode rending the filters mentioned previously with Compound Discoverer. Compounds that were not identified by the ChemSpider databases were eliminated to reduce probable misidentified compounds, obtaining 106 final compounds. Their spectra and chromatograms were revised independently, and their structures were checked according to the type of compounds (coformulants), identifying a total of 92 coformulants. Among them, 22 coformulants were identified for the first time in these samples by unknown analysis (Table 1), whereas the rest of them (70 compounds) matched with those previously detected through the analysis of suspect screening (Table 1). The fragment ions of 19 coformulants identified for the first time in these formulations were found and were confirmed with the fragments obtained by Mass Frontier. For instance, *N*-lauryldiethanolamine was identified with a retention time of 21.045 min and *m*/*z* 274.2735 [M + H]^+^, which has an abundant fragment at *m*/*z* 256.26349 (C_16_H_34_NO^+^), which is derived from the loss of a hydroxyl group. Palmitamide was identified at 2.87 min with a precursor ion at *m*/*z* 256.26349 [M + H]^+^ and fragment ions at *m*/*z* 88.07569 (C_4_H_10_NO^+^) and 102.09134 (C_5_H_12_NO^+^). The most abundant was the first one at *m*/*z* 88.07569, which is 1-aminobutan-2-ol obtained from the loss of a tridecyl group. Diethylene glycol *n*-butyl ether was detected in Kabuto JED and Duaxo at *m*/*z* 163.13252 [M + H]^+^ with a retention time of 1.855 min. This molecule possessed a fragment ion at *m*/*z* 73.06479 (C_4_H_9_O^+^). 9,12-Octadecadienamide was detected in 7 PPPs, the mass error at *m*/*z* 280.2627 was 1.973 ppm in Score 25, and the fragment ion at *m*/*z* 245.22638 (C_18_H_29_^+^) corresponds with the loss of the amide group. Oleic acid was detected with a retention time of 1.433 min at *m*/*z* 281.2484 [M – H]^−^ and the most abundant fragment ion was obtained at *m*/*z* 279.2329 as a result of a double bond between C2–C3. 2-Amino-1,3,4-octadecanetriol possessed an abundant fragment ion at *m*/*z* 300.2897 (C_18_H_38_NO_2_) [M + H]^+^ that was derived from the protonation of a hydroxyl group and it was obtained from the loss of water (H_2_O). 4-Octylbenzenesulfonic acid at *m*/*z* 269.1215 [M – H]^−^ possessed a fragment ion at *m*/*z* 79.9574. Linoleic acid was detected at *m*/*z* 279.2327 [M – H]^−^ and had a fragment ion at *m*/*z* 261.2224 (C_18_H_29_O^–^). Diethanolamine was detected at *m*/*z* 106.0864 [M + H]^+^ with an abundant fragment ion at *m*/*z* 88.0757 (C_4_H_11_ON^+^), which is obtained from the loss of a hydroxyl group. Apart from these new coformulants, three ethylene glycols were identified as [M + NH_4_]^+^ adducts. These were octaethylene glycol monohexadecyl ether, hexaethylene glycol monohexadecyl ether, and hexadecyl pentaethylene glycol ether. These were detected at *m*/*z* 612.5028, 524.4508, and 480.4245 [M + NH_4_]^+^, respectively, with a retention time of 2.28 min in Score 25. These compounds possessed a common abundant fragment at *m*/*z* 89.05971 (C_4_H_9_O_2_^+^), which corresponded with ethoxyethanol (Table 1).

**Table 1:** Identification of New Coformulants by Unknown Analyses in PPPs^a^,^b^

					characteristic ions		fragment ions				
no.	compound name	molecular formula	retention time	adduct	theoretical mass	mass error (ppm)	theoretical mass	molecular formula	mass error (ppm)	commercial product^b^	level of confidence
1	***N*-lauryldiethanolamine**	C_16_H_35_NO_2_	21.04	[M + H]^+^						all except for P15 and P16	1
							256.2635	C_16_H_34_NO	2.532		
2	diethylene glycol *n*-butyl ether	C_8_H_18_O_3_	1.86	[M + H]^+^	163.1325	–2.261	73.0648	C_4_H_9_O	7.764	P2, P6, P7, P13	2
3	9,12-octadecadienamide	C_18_H_33_NO	1.97	[M + H]^+^	280.2627	–2.340	81.0699	C_6_H_9_	5.589	P1–P7	2
							95.0855	C_7_H_11_	3.503		
							109.1012	C_8_H_13_	0.761		
							245.2264	C_18_H_29_	–1.988		
4	oleic acid	C_18_H_34_O_2_	1.43	[M – H]^−^	281.2484	–0.696	279.2330	C_18_H_31_O_2_	3.879	P1, P2, P8, P9, P13, P15, P17, P20	2
5	2-amino-1,3,4-octadecanetriol	C_18_H_39_NO_3_	19.83	[M + H]^+^	318.3000	–0.953	300.2897	C_18_H_38_NO_2_	–2.51	all except for P8 and P12	2
6	palmitoleoylglycine	C_18_H_33_NO_3_	1.94	[M + H]^+^	312.2524	–1.862	292.2271	C_18_H_30_NO_2_	4.087	P3, P6	2
							294.2428	C_18_H_32_NO_2_	5.214		
7	dodecyl 4-methylbenzenesulfonate	C_19_ H_32_O_3_S	1.27	[M – H]^−^	339.1996	–0.863	79.9574	SO_3_	–2.578	P6, P7	2
							183.0121	C_8_H_7_O_3_S	–2.561		
8	2-bromo-4,5,6,7-tetraiodo-1*H*-benzimidazole	C_7_HBrI_4_N_2_	2.41	[M + H – H_2_O]^+^	682.5444	–3.613				P3, P6, P7, P12, P14	4
			19.98	[M + H]^+^	700.5551	–3.388					
9	4-octylbenzenesulfonic acid	C_14_H_22_O_3_S	1.31	[M – H]^−^	269.1215	3.784	79.9574	O_3_S^–^	–5.204	P2, P3, P6, P7	2
10	diethoxyethylamine	C_6_H_15_NO_2_	2.61	[M + H]^+^	134.1174	–0.532				P1, P3–P7	4
11	linoleic acid	C_18_H_32_O_2_	1.45	[M – H]^−^	279.2327	–0.820	261.2224	C_18_H_29_O	3.705	P2, P3, P7	2
12	octyl 4-methylbenzenesulfonate	C_15_H_24_ O_3_S	1.31	[M – H]^−^	283.1371	–0.691	79.9574	SO_3_	–1.702	P2, P3, P6, P7	2
13	**palmitamide**	C_16_ H_34_NO	2.87	[M + H]^+^	256.2635	–2.619	88.0757	C_4_H_10_NO	3.628	All except for P3, P4, P6, P7, P12	1
							102.0913	C_5_H_12_NO	1.660		
14	phenyl 1-pentadecanesulfonate	C_21_H_36_O_3_S	1.28	[M – H]^−^	367.2308	–1.138	183.0121	C_8_H_7_O_3_S	2.452	P2, P3, P6, P7	2
15	1-acetyl-1-cyclohexene	C_8_H_12_O	1.69	[M + NH_4_]^+^	142.1222	–3.310	95.04914	C_6_H_7_O	3.457	P3, P7	2
							71.04914	C_4_H_7_O	8.003		
16	*N*-(20-amino-4-{3-[(octylsulfonyl)amino]propyl}-4,8,12,17-tetraazaicos-1-yl)-1-octanesulfonamide	C_35_H_79_N_7_O_4_S_2_	20.28	[M + H]^+^	726.5709	0.161				P3, P6, P7	4
17	2-(2-(2-(dodecyloxy)-ethoxy)-ethoxy)-acetamide	C_18_ H_37_ N O_4_	2.53	[M + H]^+^	332.2786	–2.273	70.04132	C_4_H_6_O	7.657	P1–P7	2
							97.10198	C_7_H_13_	2.503		
18	C16 phytosphingosine	C_16_H_35_ NO_3_	2.69	[M + H]^+^	290.2682	–2.513	74.0600	C_3_H_8_ON	7.285	P1–P7, P15–P20	2
							122.0812	C_4_H_12_O_3_N	–0.244		
							242.2478	C_15_H_32_ON	–2.440		
19	diethanolamine	C_4_H_11_NO_2_	7.77	[M + H]^+^	106.0864	1.422	88.0757	C_4_H_10_NO	6.580	P1–P7	2
20	1-dibutylamino-3-methoxy-propan-2-ol	C_12_H_27_NO_2_	19.27	[M + H]^+^	218.2110	–2.200	106.0863	C_4_H_12_O_2_N	1.742	P1–P7	2
							200.2009	C_12_H_26_ON	–1.853		
21	3,6,9,12-tetraoxaoctacosan-1-ol	C_24_H_50_O_5_	20.34	[M + NH_4_]^+^	436.3987	–2.377	89.0597	C_4_H_9_O_2_	5.209	P1–P7, P12	2
							133.0859	C_6_H_13_O_3_	–1.133		
22	linoleamide	C_18_H_33_NO	18.50	[M + H]^+^	280.2629	–2.210	81.0699	C_6_H_9_	4.849	P1–P14	2
							95.0855	C_7_H_11_	2.346		
							109.1012	C_8_H_13_	0.394		
							263.2369	C_16_H_29_N_3_	1.598		

a Compounds in bold were confirmed with their analytical standard.

b Commercial product abbreviation indicated in Section 2.1.

### 3.2 Confirmation of Coformulants

**Figure 2:**
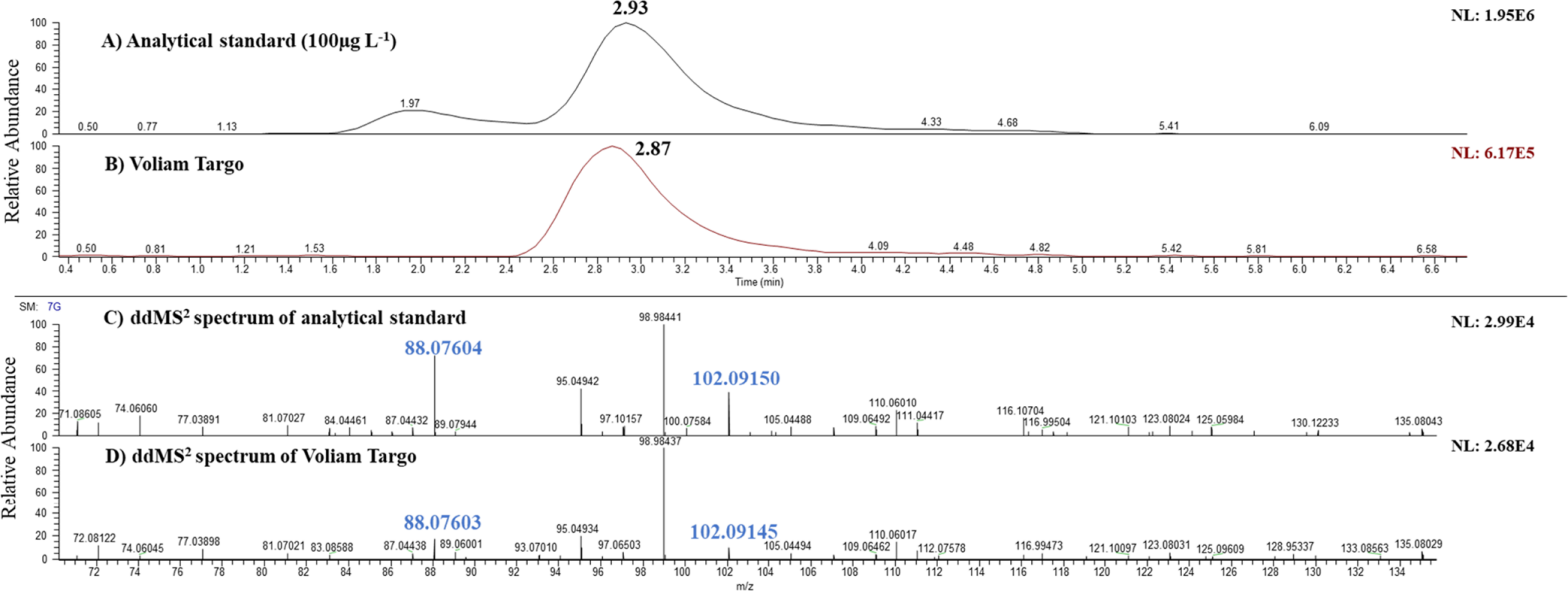
Extracted ion chromatograms and spectra of palmitamide: (A) analytical standard at 100 μg/L, (B) Voliam Targo commercial product, (C) ddMS^2^ spectrum of the analytical standard, and (D) ddMS^2^ spectrum of Voliam Targo.

Taking into account the coformulants detected in a greater number of the analyzed PPPs, in addition to peaks with a high intensity as well as availability at the time of the study, 14 analytical standards were purchased to confirm them. These were 4-dodecylbenzenosulfonic acid, 1-naphthalenesulfonic acid, triethylene glycol monomethyl ether, *N*,*N*-dimethyldecanamide, 4-*s*-butyl-2,6-di-*tert*-butylphenol, lauramide DEA, 1-ethyl-2-pyrrolidone, sorbitan monostearate, 2,6-dimethylaniline, aniline, *N*-lauryldiethanolamine, palmitamide, xylene, and metilox. These standards were injected into LC-Q-Orbitrap-MS. The spectrum of each analytical standard was compared with those obtained in the samples. Twelve standards were verified in the samples taking into account the *m*/*z* values of precursor ions and their fragments reported during their tentative identification (Table S5) and also by comparing the ion ratio shown in Table 2. In addition, these compounds were confirmed by the retention time, and the time shift was lower than ±0.1 min. On the other hand, the retention time of the standards xylene and metilox did not match with those obtained in the samples; thus, the two unknown peaks were misidentified as xylene and metilox. As a result, the research methodology used in this study was successful because 85.7% of the acquired compounds were verified in the analyzed samples. For example, Figure 2 shows the extracted ion chromatogram (EIC) of palmitamide [M + H]^+^ in the analytical standard (100 μg/mL) (a), in Voliam Targo (b) and (c) ddMS^2^ spectrum of the analytical standard, and (d) ddMS^2^ spectrum of the Voliam Targo. The confirmation of this compound was chosen based on the matching MS spectra. The retention time shift was 0.06 min, which was less than ±0.1 min. The characteristic ion at *m*/*z* 256.2635 had a mass error of −2.619 ppm. ddMS^2^ spectra also showed a highly matching pattern. Fragments at *m*/*z* 88.0757 and 102.0913 had mass errors of 3.628 and 1.660 ppm, respectively. Nevertheless, smaller differences in the RT, peak shape, and ddMS^2^ spectra could be due to the matrix interferences in the standard, whose purity is higher than 95%. The standard 4-*s*-butyl-2,6-di-*tert*-butylphenol was only detected in the negative mode. In addition, the fragment ions from the standard 4-*s*-butyl-2,6-di-*tert*-butylphenol were not found in its negative mode in the Mass Frontier and in the literature. For this reason, this compound was confirmed by comparison of the retention time of the peak obtained by EIC of the commercial product with the analytical standard and by comparison of its full-scan mass spectra with the theoretical one.

**Table 2:** Analytical Parameters of the Analytical Method

					%RSD (*n* = 5) at 50 μg/L			
compounds	linear range (μg/L)	calibration curve (μg/L)	linearity (*R*^2^)	LOQ(mg/L)	intraday	interday	matrix effect (%)	fragments (ion ratio)
dodecylbenzenosulfonic acid	LOQ-100	*y* = 6.2169*x* + 0.3504	0.9940	0.004	3.7	7.8	40–108	183.0121 (100%)
								79.9574 (7.2%)
1-naphthalenesulfonic acid	LOQ-100	*y* = 17.375*x* - 0.4666	0.9951	0.0002	2.6	3.5	42–160	143.0502 (100%)
								79.9574 (45.0%)
triethylene glycol monomethyl ether	LOQ-100	*y* = 22.011*x* + 0.0033	0.9990	0.0002	0.4	2.2	28–103	103.0754 (100%)
*N*,*N*-dimethyldecanamide	LOQ-100	*y* = 51.782*x* + 1.8206	0.9963	0.001	1.6	5.2	1.1–115	198.1852 (100%)
								116.1070 (15.6%)
								102.0913 (10.3%)
								130.1264 (5.7%)
4-*sec*-butyl-2,6-di-*tert*-butylphenol	LOQ-100	*y* = 0.0441*x* - 0.1513	0.9978	0.001	0.2	9.1	67–70	245.2264 (100%)
								207.1742 (19.8%)
lauramide DEA	LOQ-50	*y* = 14.909*x* + 0.1623	0.9985	0.0001	2.3	3.1	38–74	106.0863 (100%)
								70.0651 (54.9%)
								88.0766 (27.3%)
1-ethyl-2-pyrrolidone	LOQ-100	*y* = 16.186*x* + 0.1392	0.9983	0.0001	3.6	8.9	39–83	112.0757 (100%)
sorbitan monostearate	LOQ-100	*y* = 0.0802*x* – 0.0548	0.9900	0.003	7.3	9.0	84–95	279.2330 (100%)
								59.0126 (1.5%)
2,6-dimethylaniline	LOQ-100	*y* = 2.0645*x* + 0.1093	0.9980	0.0001	2.2	9.6	56–105	107.0731(100%)
								105.0699 (50.7%)
aniline	LOQ-100	*y* = 0.2907*x* + 0.2334	0.9931	0.001	4.2	5.6	46–113	-
palmitamide	LOQ-100	*y* = 0.5792*x* + 0.061	0.9973	0.0003	0.2	4.0	9–84	102.0913 (100%)
								88.0757 (56.0%)
*N*-lauryldiethanolamine	LOQ-100	*y* = 67.849*x* – 3.3062	0.9995	0.001	2.0	4.9	62–80	90.05495 (100%)
								256.2635 (50.5%)

A literature search was carried out to explain the role of each coformulant after their confirmation by analytical standards, showing a summary of these properties in Table S6. Dodecylbenzenesulfonic acid and 1-naphthalenesulfonic acid are anionic surfactants very often used in PPPs to clean different surfaces due to their good dispersing, emulsifying, wetting, and foaming properties.^25,26^ Triethylene glycol monomethyl ether is an alkyl glycol ether used as a solvent in PPPs due to its high solvency glycol ether with excellent coupling properties.^27^*N*,*N*-Dimethyldecanamide is used as a solvent for active ingredients in agricultural formulations.^13^ 4-*sec*-Butyl-2,6-di-*tert*-butylphenol is an analogue of 2,4-di-*tert*-butylphenol that may be used as a preservative in nontoxic aqueous pesticides.^28^ This compound has been identified in the PPPs Kabuto Jed.^2^ Lauramide DEA is a common thickening, foam enhancer, and stabilizer in cosmetics and shampoos, and this compound has been previously detected in Altacor formulation.^2^ 1-Ethyl-2-pyrrolodine is an aprotic solvent, which is used worldwide due to its water solubility and solvent power, and it is used for different applications, including pesticides, the pharmaceutical industry, and cosmetic products.^29^ Span-60 is a nonionic surfactant used as an emulsifier and a stabilizer agent in medicine, cosmetic, food, pesticide, coating, plastic, and textiles industries.^30^ 2,6-Dimethylaniline is used as a chemical intermediate in the manufacture of pesticides.^31^ In addition, it has been stated that aniline is used in agricultural fungicides and herbicides, and this substance has been identified previously in Voliam Targo and Altacor.^2^ Palmitamide is a nonionic surfactant derived from the palm oil.^32^*N*-Lauryldiethanolamine is an antistatic agent and cosmetic ingredient that belongs to the class of ionizable surfactants.^33^

### 3.3 Quantification of Coformulants in the Commercial Samples

Table 2 lists the analytical parameters of the method used, including the linear range, calibration curve, coefficient of determination, LOQ, ME, intra- and interday precision (%RSD), the *m*/*z* of fragment ions, and their ion ratio. All calibration curves showed good linearity and the determination coefficients were higher than 0.9900. The LOQ was the lowest concentration of each compound that was possible to determine in the PPP samples after their dilutions. The LOQ was assessed by reference points in the solvent at low concentrations, choosing as the LOQ the concentration that achieves acceptable results in terms of precision (RSD < 20%) and linearity (determination coefficients were higher than 0.9900). The LOQ was 0.0001–0.004 mg/L.

A signal enhancement was observed in most of the compounds (ME higher than 20%) with the exception of *N*,*N*-dimethyldecanamide in Massocur and palmitamide in Voliam Targo that did not show a matrix effect (1 and 9% of ME). Therefore, the standard addition methodology was used in order to estimate the concentration of the detected compounds in the PPPs. A negligible matrix effect was estimated for *N*,*N*-dimethyldecanamide and palmitamide, and it may be explained due to the fact that both are alkyl amides; it may be that these types of compounds are less influenced by the interfering substances in these phytosanitary products. In addition, these compounds may have been added in a greater proportion in Massocur and Voliam Targo than in the rest of PPPs. Intraday precision (% RSD) at 50 μg/L was lower than 7.3% in all cases, and interday precision was lower than 9.6%. The interday precision was similar to the reported previously by the C18 column at 100 μg/L, which was lower than 8% in all cases.^2^ Additionally, recoveries were also evaluated, and they ranged between 88 and 118% (Table S7).

Table 3 shows that the concentrations of coformulants found in the studied PPPs ranged from 0.001 g/L (Lauramide DEA, 1-ethyl-2-pyrrolidone and 2,6-dimethylaniline) to 68.78 g/L (dodecylbenzenesulfonic acid). The most concentrated coformulants in most of PPPs was dodecylbenzenesulfonic acid, at concentration values ranging from 0.03 g/L in Flint Max (WC) to 68.78 g/L in Score 25 (EC). Maldonado-Reina et al. reported concentration values in dodecylbenzenesulfonic acid in Kabuto JED, Mavita, Lexor 25, Score 25, Ampligo, Nomada, and Duaxo (11.67, 32.33, 28.15. 28.3, 0.83, 16.93, and 10.35 g/L respectively),^2^ which were in the same order as obtained in the current study. This compound was also compared with the safety data sheets since it is the only coformulant whose concentration is reported. The content of this surfactant was within the range according to their safety data sheets of Lexor 25 (10–50 g/L), Score 25 (30–100 g/L), and Nomada (<50 g/L). Nevertheless, the content in PPPs was much lower than that reported by the safe data sheets in Mavita (≥0–50 g/L), Latino (14 g/L), and Impala Star (<10 g/L). The second most concentrated coformulant in most PPPs was aniline, which ranged from 0.027 g/L in Latino (EC) to 0.726 g/L in Ampligo (ZC). In the case of Altacor (WG), its aniline concentration (0.874 g/kg) was in the same order of magnitude that was obtained in a previous study.^2^ In addition, the content of *N*,*N*-dimethyldecanamide ranged from 0.011 g/L obtained in Topas (EW) to 1.623 g/L in Massocur 12.5 (EC). The content obtained in Massocur was in the same order of magnitude as that reported by Hergueta-Castillo et al., which was 1.84 g/L.^14^ Palmitamide and sorbitan monostearate are also concentrated coformulants in most formulations, the highest content of palmitamide being in Voliam Targo (SC) (0.615 g/L), whereas Score 25 (EC) possessed the highest content in sorbitan monostearate (0.486 g/L). *N*-Lauryldiethanolamine was found in all PPPs but at concentrations lower than 0.059 g/L. In addition, naphthalenesulfonic acid was quantified in 15 PPPs. This compound was previously quantified in Altacor (WG) at a similar content, 0.196 g/kg^2^. Ethyl-pyrrolidin-2-one was found between 0.001 g/L in Impala Star (EW) and 0.113 g/L in Duaxo (EC). In contrast, triethylene glycol monomethyl ether and 4-*s*-butyl-2,6-di-*tert*-butylphenol were found below 0.025 g/L. In addition, the concentration of 2,6-dimethylaniline was found to be lower than 0.003 g/L. A wide variation in the coformulant contents in PPPs could be observed, which depends on the brand of PPPs.

**Table 3:** Quantification of Coformulants in PPP^a^

	dodecylbenzenesulfonic acid	1-naphthalenesulfonic acid	triethylene glycol monomethyl ether	*N*,*N*-dimethyldecanamide	4-*s*-butyl-2,6-di-*tert*-butylphenol	lauramide DEA	1-ethyl-2-pyrrolidone	sorbitan monostearate	2,6-dimethylaniline	aniline	palmitamide	*N*-lauryldiethanolamine
Voliam Targo	0.043	0.006				0.001	0.103			0.718	0.615	0.010
Kabuto JED	6.649		0.015			0.121		0.199			0.018	0.013
Mavita	19.768	0.015										0.012
Cidely Top	0.094	0.002		0.142			0.025					0.012
Dynali	0.127	0.004		0.236		0.001					0.032	0.020
Lexor 25	25.284		0.007					0.051				0.014
Score 25	68.784				0.020			0.486				0.010
Dagonis	0.132	0.008						0.036	0.003		0.051	0.012
Coragen 20 SC	0.127	0.008	0.003			0.002				0.565	0.012	0.022
Altacor 2	0.110	0.196	0.002			0.005			0.005	0.874	0.003	0.015
Ampligo	0.319	0.006	0.003			0.001				0.726	0.014	0.016
Nomada	14.575				0.010			0.091				0.013
Duaxo	9.358	0.010	0.019			0.001	0.113	0.150			0.037	0.026
Ortiva Top	0.069	0.772	0.015				0.003				0.043	0.014
Flint Max	0.031	0.340			0.025							
Topas	0.223	0.161	0.007	0.011								
Massocur 12.5 EC	6.338		0.016	1.623		0.001	0.002				0.029	0.059
Impact Evo	0.091	0.418	0.006				0.007				0.028	0.014
Latino (Mitrus)	2.835	0.006			0.022		0.002	0.175	0.001	0.027	0.052	0.017
Impala Star	1.416	0.010				0.003	0.001			0.198	0.044	0.021

a Results are expressed in g/L; Altacor and Flint Max results are expressed in g/kg.

### 3.4 Toxicity

Toxicological information on coformulants used in these PPPs is required to assess whether these chemical substances affect human health.

**Figure 3:**
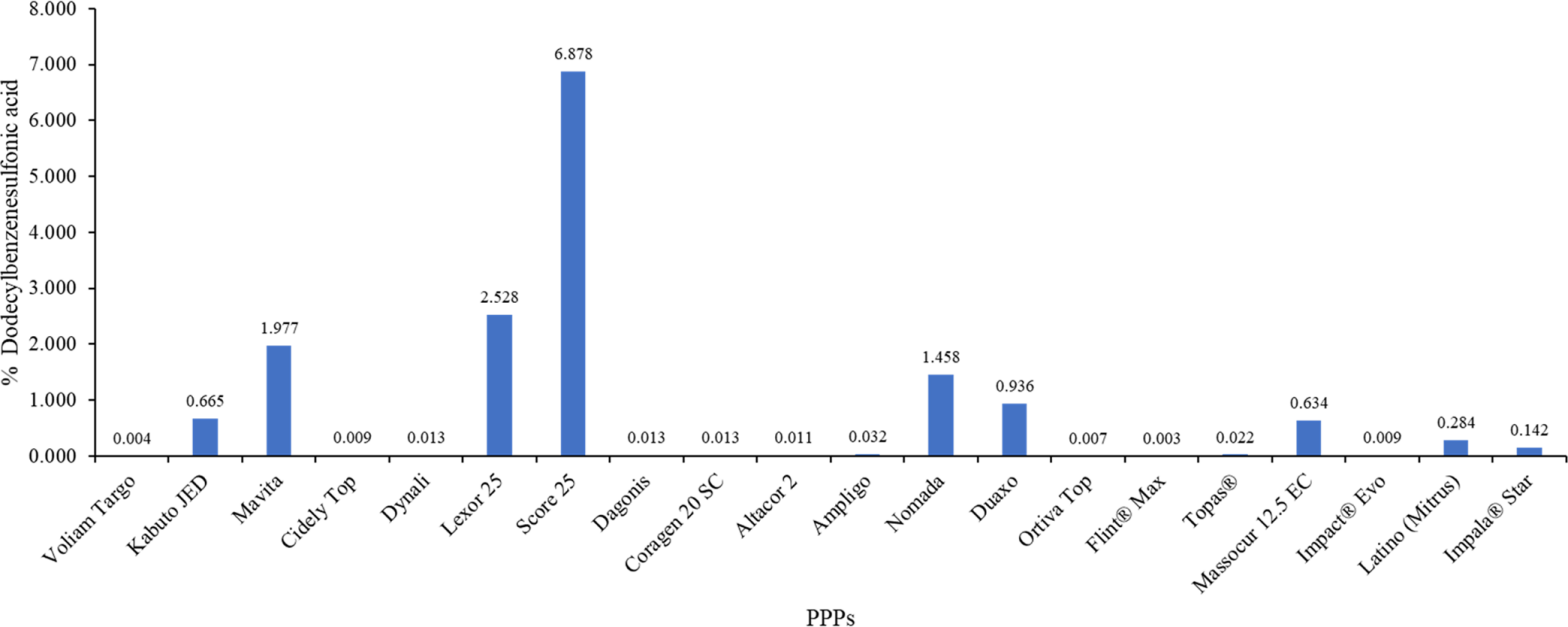
Content of dodecylbenzenesulfonic acid in PPPs. All results are expressed in % (w/v). Altacor and Flint Max are expressed as % (*w/w*).

Alkyl benzenesulfonates and alkyl naphthalenesulfonates possess an oral reference dose (RfD) value of 0.5 mg/kg/day. Dodecylbenzenesulfonic acid was obtained in high content in PPPs, reaching 6.878% (w/v) in Score 25 (Figure 3). Nevertheless, aniline and *N*,*N*-dimethylaniline present higher toxicity in comparison with dodecylbenzenesulfonic acid RfD (0.007 and 0.003 mg/kg/day). Finally, no information regarding their RfD was found for 1-ethyl-2-pyrrolidone, lauramide DEA, *N*,*N*-dimethyldecanamide, and triethylene glycol monomethyl ether.

The median lethal dose (LD_50_) is the amount of a substance that causes the death of 50% of a group of test animals. The Toxicity Estimation Software Tool (T.E.S.T), which is an open-source application developed by the US EPA, estimates the LD_50_ of a compound by applying several methodologies to have greater confidence in predicted toxicities. Table 4 shows the LD_50_ obtained by the use of the Toxicity Estimation Software Tool (T.E.S.T). Among them, aniline is the most toxic compound identified in this study due to its low value of LD_50_ (0.372 g/kg). In addition, *N*,*N*-dimethyldecanamide has a low LD_50_ and it is classified as harmful to aquatic life with long-lasting effects, causing serious eye irritation and skin irritation, and may cause respiratory irritation.^34^ By comparison of the LD_50_ of these coformulants with the active ingredients that composed the PPPs, it was observed that aniline was detected in PPPs that are composed of chlorantraniliprole, lambda-cyhalothrin, myclobutanil, and fenbuconazole. This coformulant possesses an LD_50_ lower than chlorantraniliprole (2.555 g/kg) and fenbuconazole (1.174 g/kg). Nevertheless, lambda-cyhalothrin (0.369 g/kg) and myclobutanil (0.166 g/kg) possess a lower toxicity than aniline. In addition, dimethylaniline, dodecylbenzenesulfonic acid, and ethyl-pyrrolidin-2-one possess a LD_50_ lower than chlorantraniliprole (2.555 g/kg) and tebuconazole (3.120 g/kg).

**Table 4:** Toxicological Information of Confirmed Coformulants

coformulant	median Lethal Dose (LD_50_) (T.E.S.T. g/kg)	class (Toxtree)^a^
aniline	0.372	III
1-naphthalenesulfonic acid	4.873	III
dodecylbenzenesulfonic acid	1.297	I
1-ethyl-2-pyrrolidone	1.44	III
*N*,*N*-dimethylaniline	0.78	I
lauramide DEA	8.175	III
*N*,*N*-dimethyldecanamide	4.395	III
triethylene glycol monomethyl ether	10.967	I
sorbitan monostearate	28.396	III
4-*s*-butyl-2.6-di-*tert*-butylphenol	15.85	II
*N*-lauryldiethanolamine	6.599	I
palmitamide	3.682	I

a Toxtree: Toxic hazard estimation by decision tree approach (Toxtree).

The toxicity of coformulants by using Toxic hazard estimation by decision tree approach (Toxtree) according to the Cramer rules was also included in Table 4. This approach classifies organic chemicals into one of three classes (I for low, II for intermediate and III for high, i.e., Cramer classes) reflecting the probability of low, moderate, and high toxicity in an explicit way.^35^ It should be noted that active ingredients in PPPs belong to class III due to their high toxicity. Nevertheless, aniline, 1-naphthalenesulfonic acid, 1-ethyl-2-pyrrolidone, *N*,*N*-dimethyldecanamide, lauramide DEA, and sorbitan monostearate have also high toxicity (class III). For all of these reasons, the content of these types of coformulants in PPPs should be controlled to avoid adverse effects on health. In fact, regulation (EU) 2021/383 of the Commission of March 3, 2021, established that aniline, 2-pyrrolidone, and naphthalene are unacceptable coformulants for inclusion in PPP in Spain because these are carcinogenic and toxic to reproduction.^9^

In summary, it is stated that this new method based on the use of a polyhydroxy methacrylate stationary phase with LC-HRMS was effective for the tentative identification of 92 coformulants in PPPs. Among them, 48 compounds were detected for the first time in the target 20 PPPs (26 were detected by the suspect strategy and confirmed by unknown analysis, whereas 22 new coformulants were identified by unknown analyses). These compounds may be mainly classified in anionic surfactants, such as sulfates of ethylene glycol alkyl ethers and alkyl benzenes, amphoteric surfactants, and other nonionic surfactants, including alkyl phenoxyethanols, alkyl alcohols, ethoxy ethyl amines, ethanol amines, amino alcohols, ethylene glycol ether, fatty amides, fatty acids such as oleic acid, and other compounds.

Furthermore, the methodology based on LC-HRMS has allowed the confirmation as well as the quantification of 12 compounds after the acquisition of standards. Dodecylbenzenesulfonic acid was the most concentrated compound in most formulations, with Score 25 containing the highest proportion of this coformulant at 6.87% (w/v). In addition, triethylene glycol monomethyl ether, 4-*s*-butyl-2,6-di-*tert-*butylphenol, 1-ethyl-2-pyrrolidone, sorbitan monostearate, 2,6-dimethylaniline, palmitamide, and N-lauryldiethanolamine have been quantified for the first time in these PPPs. Finally, it will be important to consider the toxicity of these coformulants since aniline, naphthalenesulfonic acid, 1-ethyl-2-pyrrolidone, *N*,*N*-dimethyldecanamide, lauramide DEA, and sorbitan monostearate have high toxicity, which could have adverse effects on health. Therefore, this method could be further developed to determine possible residues derived from coformulants found in phytosanitary products in crops. In addition, previous studies have shown that the C18 column is more suitable for the separation of nonionic surfactants, such as thiazoles, poly(ethylene oxide) with long chains, alkyl naphthalene, and other certain hydrophobic compounds, such as monopalmitin and glyceryl monosterate. Nevertheless, these compounds were not detected by the method developed with the Shodex column. Therefore, for a comprehensive characterization of coformulants in PPPs, a complementary use of both polymer-based and C18 stationary phases would be necessary.

## Supporting Information Available

Analyzed plant protection products (Table S1); gradient conditions for the method employed for the Acclaim Surfactant Plus column (Table S2); compounds included in the homemade database (Table S3); tentative identification of coformulants by the Acclaim Surfactant Plus column (Table S4); identification of coformulants by suspect analyses in PPPs; compounds in bold were confirmed with their analytical standard (Table S5); properties of coformulants confirmed in PPPs (Table S6); evaluation of coformulant recoveries (spiked concentration: 50 μg/L) in Kabuto JED (Table S7); and extracted ion chromatograms and spectra of octyl 4-methylbenzenesulfonate: (A) Lexor with the Shodex column, (B) Lexor with the Acclaim column, (C) full MS spectrum with the Shodex column, (D) full MS spectrum with Acclaim, and (E) theoretical full MS spectrum (Figure S1) (PDF)
