# Supplementary material for: Unveiling Coformulants in Plant Protection Products by LC-HRMS Using a Polyhydroxy Methacrylate Stationary Phase
Source: J Agric Food Chem. 2023 Oct 17;71(42):15842–54. doi: 10.1021/acs.jafc.3c03600 (PMC10603805; doi:10.1021/acs.jafc.3c03600)

SUPPLEMENTARY MATERIAL to the article: Unveiling co-formulants in plant protection products by LC-HRMS using a polyhydroxy methacrylate stationary phase

Beatriz Martín-García, Roberto Romero-González*, José Luis Martínez Vidal, Antonia Garrido Frenich

Research group "Analytical Chemistry of Contaminants", Department of Chemistry and Physics, Research Centre for Mediterranean Intensive Agrosystems and Agri-Food Biotechnology (CIAMBITAL), University of Almería, Agri-Food Campus of International Excellence, ceiA3, 04120, Almería, Spain.

*Corresponding author: rromero@ual.es

ORCID codes

Beatriz Martín García: 0000-0002-6367-1333

Roberto Romero-González: 0000-0002-2505-2056

José Luis Martínez Vidal: 0000-0003-0655-2597

Antonia Garrido-Frenich: 0000-0002-7904-7842

Table S1. Analysed Plant Protection Products.[a]

Abbreviation	Commercial name	Formulation	Manufacturer	Composition (*w/v*)	Supplier
**P1**	Voliam® targo	SC	Syngenta crop protection ag	4.5 % chlorantraniliprole	Voliam® targo
**P2**	Kabuto® JED	EC	Kenogard	1.67 % Difenoconazole	Proag España S.L. (Villacarrillo, Spain)
**P3**	Mavita® 250	EC	Adama	25 % Difenoconazole	Agro 21 S.L. (La Almunia de Doña Godina, Spain)
**P4**	Cidely® Top	DC	Syngenta Crop Protection AG	12.5 % Difenoconazole	Suministros Agrícolas Hnos. López S.A (La Mojonera, Almería)
**P5**	Dynali®	DC	Syngenta Crop Protection AG	6 % Difenoconazole 3 % Ciflufenamid	Nueva Pangea S.L. (Valdepeñas, Spain)
**P6**	Lexor-25	EC	Syngenta Crop Protection AG	25 % Difenoconazole	Jardinia Productos de Jardín S.L. (Elche, Spain)
**P7**	Score® 25	EC	Syngenta Crop Protection AG	25 % Difenoconazole	Suministros Agrícolas Hnos. López S.A (La Mojonera, Almería)
**P8**	Dagonis®	SC	BASF Agro B.V.	7.5 % Fluxapyroxad 5 % Difenoconazole	Suministros Agrícolas Hnos. López S.A (La Mojonera, Almería)
**P9**	Coragen® 20	SC	FMC International Switzerland	20 % Chlorantraniliprole	Sercopag Asesoría Agrícola (Bolanos de Calatrava, Spain)
**P10**	Altacor® 35	WG	FMC International Switzerland	35 % Chlorantraniliprole (*w/w*)	Suministros Agrícolas Hnos. López S.A (La Mojonera, Almería)
**P11**	Ampligo® 150 ZC	ZC	Syngenta Crop Protection AG	10 % Chlorantraniliprole 5 % Lambda-Cyhalothrin	Fito Agrícola S.L. (Castellón, Spain)
**P12**	Nomada	EC	Globachem N.V.	25 % Difenoconazole	Fito Agrícola S.L. (Castellón, Spain)
**P13**	Duaxo®	EC	COMPO GmbH	1.67 % Difenoconazole	Leroy Merlin (Lezennes, France)
**P14**	Ortiva® Top	SC	Syngenta Crop Protection AG	20 % Azoxystrobin 12.5 % Difenoconazole	Suministros Agrícolas Hnos. López S.A (La Mojonera, Almería)
**P15**	Flint® Max	WG	BAYER AG (Leverkusen,	50% tebuconazole (*w/w*)	Bayer Cropscience, S.L. (Barcelona, Spain)

			Germany)		
**P16**	Topas®	EW	Syngenta Crop Protection AG (Basilea, Switzerland)	19.4% penconazole (*w/w*)	Syngenta España, S.A. (Madrid, Spain)
**P17**	Massocur 12.5	EC	Sharda Cropchem Ltd. (Mumbai, India)	12.5% myclobutanil	Sharda Europe Bvba (Asse, Belgium)
**P18**	Impact® Evo	SC	Cheminova A/S (Lemvig, Denmark)	12.5% flutriafol	FMC Agricultural Solutions, S.A.U. (Madrid, Spain)
**P19**	Latino (Mitrus)	EC	Industrias Afrasa, S.A. (Valencia, Spain)	12.5% myclobutanil	Industrias Afrasa, S.A. (Valencia, Spain)
**P20**	Impala® Star	EW	Corteva Agriscience Spain, S.L.U. (Sevilla, Spain)	2.5% fenbuconazole	Corteva Agriscience Spain, S.L.U. (Sevilla, Spain)

[a] Abbreviation: DC: dispersible concentrate; EC: emulsifiable concentrate; SC: suspension concentrate; WG: wettable granules; ZC: a mixture of capsule suspension (CS) in SC; EW: emulsion, oil in water; WG: water dispersible granule.

Table S2. Gradient conditions for the method employed for Acclaim Surfactant Plus column.

Time (min)	Flow (mL/min)	% A	% B	%C
0	0.3	65	5	30
1	0.3	65	5	30
8	0.3	10	5	85
21	0.3	10	5	85
30	0.3	65	5	85

A: Water, B: 100 mM Ammonium acetate, at pH 5, C: Acetonitrile

Table S3. Compounds included in the homemade database

Compound Name	Molecular ion (*m/z*)	Precursor ion (*m/z*)	Adduct
1,2-Benzisothiazol-3(2H)-one	151.0086	152.0165	$[M+H]^+$
1,4-Dioxane	88.0519	89.0597	$[M+H]^+$
1- monopalmitin	330.2765	331.2843	$[M+H]^+$
1-Butyl-1-naphthalenesulfonic acid	264.0826	263.0747	$[M+H]^+$
1-Dodecanol	186.1978	187.2056	$[M+H]^+$
1-Dodecylnaphthalene	296.2499	297.2577	$[M+H]^+$
1-Ethylpyrrolidin-2-one	113.0835	114.0913	$[M+H]^+$
1-Hexadecylnaphthalene	352.3125	353.3203	$[M+H]^+$
1-Methoxy-2-propanol	90.0675	91.0754	$[M+H]^+$
1-Methyl-2,6-cyclohexadione	126.0675	127.0754	$[M+H]^+$
1-Methylpyrrolidin-2-one	99.0679	100.0757	$[M+H]^+$
1-Naphthalenesulfonic acid	208.0200	207.0121	$[M-H]^-$
1-Naphthalenesulfonic acid	208.0200	209.0267	$[M+H]^+$
1-Nonylnaphthalene	254.2029	255.2107	$[M+H]^+$
1,3,5-Triazine-1,3,5(2H,4H,6H)-triethanol (Actane)	219.1577	220.1656	$[M+H]^+$
17-(4-Nonylphenoxy)-3,6,9,12,15-pentaoxaheptadecan-1-ol	484.3395	485.3473	$[M+H]^+$
2,6-Di-tert-butylnaphthalene-1-sulfonic acid	320.1452	319.1373	$[M+H]^+$
2-(4-Methyl-1-piperazinyl)ethanol	144.1257	145.1335	$[M+H]^+$
2-(p-Octylphenoxy)ethanol	250.1927	251.2006	$[M+H]^+$
2-[(Hydroxymethyl)amino]ethanol	91.0633	92.0706	$[M+H]^+$
2-[2-(4-Nonylphenoxy)ethoxy]ethanol	308.2346	309.2424	$[M+H]^+$
2-[2-[2-(4-Octylphenoxy)ethoxy]ethoxy]ethanol	338.2452	339.2530	$[M+H]^+$
2-[2-[2-[2-(4-Nonylphenoxy)ethoxy]ethoxy]ethoxy]ethanol	396.2870	397.2949	$[M+H]^+$
2-Amino-1,3-dimethylbenzene (2,6-Dimethylaniline)	121.0886	122.0964	$[M+H]^+$
2-Bentanol (Benzyl alcohol)	108.0570	109.0648	$[M+H]^+$
2-Butoxyethyl oleate	382.3442	383.3520	$[M+H]^+$
2-Butyl-1-naphthalenesulfonic acid	264.0826	263.0747	$[M-H]^-$
2-Dodecylnaphthalene	296.2499	297.2577	$[M+H]^+$
2-ethoxyethanol	90.0675	91.0754	$[M+H]^+$
2-Hexadecylnaphthalene	352.3125	353.3203	$[M+H]^+$
2-Methoxyethanol	76.0519	77.0597	$[M+H]^+$
2-Methoxyethyl acetate	118.0625	119.0703	$[M+H]^+$
2-Methoxypropanol	90.0675	91.0754	$[M+H]^+$
2-Methylisothiazolone	115.0086	116.0165	$[M+H]^+$
2-Naphthalenesulfonic acid	208.0200	207.0121	$[M-H]^-$
2-Nitropropane	89.0471	90.0550	$[M+H]^+$

2-Palmitoylglicerol	330.2765	331.2843	$[M+H]^+$
2-Phenylpropanal	134.0726	135.0804	$[M+H]^+$
2.2-Dimethylocta-3,4-dienal	152.1196	153.1274	$[M+H]^+$
20-(4-Nonylphenoxy)-3,6,9,12,15,18-hexaoxaicosan-1-ol	528.3657	529.3735	$[M+H]^+$
20-(4-Octylphenoxy)-3,6,9,12,15,18-hexaoxaicosan-1-ol	514.3500	515.3578	$[M+H]^+$
20-[4-(1.1.3.3-Tetramethylbutyl)phenoxy]-3,6,9,12,15,18-hexaoxaicosan-1-ol	514.3500	515.3578	$[M+H]^+$
26-(4-Nonylphenoxy)-3,6,9,12,15,18,21,24-octaoxahexacosan-1-ol	616.4181	617.4259	$[M+H]^+$
3,6,9,12,15,18,21,24,27,30,33,36-dodecaoxanonatetracontan-1-ol	728.5286	729.5359	$[M+H]^+$
3-hexenal	98.0732	99.0804	$[M+H]^+$
3.6.9.12-tetraoxapentacosan-1-ol	376.3183	377.3262	$[M+H]^+$
3.6,9,12-Tetraoxatetradecan-1-ol, 14-[4-(1,1,3,3-tetramethylbutyl)phenoxy]-	426.2976	427.3054	$[M+H]^+$
3,6,9,12,15-Pentaoxaheptadecan-1-ol, 17-(4-octylphenoxy)-	470.3238	471.3316	$[M+H]^+$
3,6,9,12,15-Pentaoxaheptadecan-1-ol, 17-[4-(1,1,3,3-tetramethylbutyl)phenoxy]-	470.3238	471.3316	$[M+H]^+$
3,6,9,12,15,18,21,24-Octaoxahexacosan-1-ol,26-(4-octylphenoxy)-	602.4025	603.4103	$[M+H]^+$
3,6,9,12,15,18,21,24-Octaoxahexacosan-1-ol,26-[4-(1,1,3,3-tetramethylbutyl)phenoxy]-	602.4025	603.4103	$[M+H]^+$
3,6,9,12,15,18,21,24,27-Nonaoxanonacosan-1-ol, 29-[4-(1,1,3,3-tetramethylbutyl)phenoxy]-	646.4287	647.4365	$[M+H]^+$
4- decyl benzenesulfonic acid	298.1608	297.1530	$[M-H]^-$
4-(1-Ethyl-1,3-dimethylpentyl)phenol	220.18217	221.1900	$[M+H]^+$
4-(2-Methyloctan-2-yl)phenol	220.1822	221.1900	$[M+H]^+$
4-(3-Methyloctan-3-yl)phenol	220.1822	221.1900	$[M+H]^+$
4-(3.6-Dimethylheptan-3-yl)phenol	220.1822	221.1900	$[M+H]^+$
4-Decylbenzenesulfonic acid	298.1608	297.1530	$[M-H]^-$
4-Dodecylbenzenesulfonic acid	326.1921	325.1843	$[M-H]^-$
4-Methoxyphenol	124.0519	125.0597	$[M+H]^+$
4-Nonylbenzenesulfonic acid	284.1452	283.1373	$[M-H]^-$
4-Nonylphenol	220.1822	221.1900	$[M+H]^+$
4-Octylphenol	206.1665	207.1743	$[M+H]^+$
4-Phenylcyclohexanone	174.1039	175.1117	$[M+H]^+$
4-Phenylsulfonic acid	173.9992	172.9914	$[M-H]^-$
4-sec-Butyl-2.6-di-tert-butylphenol	262.2291	263.2369	$[M+H]^+$
4-tert-Octylphenol	206.1665	207.1743	$[M+H]^+$

4-Tetradecylbenzenesulfonic acid	354.2234	354.2234	$[M-H]^-$
4-Tridecylbenzenesulfonic acid	340.2078	340.2078	$[M-H]^-$
4-Undecylbenzenesulfonic acid	312.1765	311.1686	$[M-H]^-$
5-Chloro-2-methyl-4-isothiazolin-3-one (Methylchloroisothiazolinone)	148.9697	149.9775	$[M+H]^+$
8-(2-Methyl-2-undecanyl)-1-naphthalenesulfonic acid	376.2078	375.1999	$[M-H]^-$
9-Octadecenamide	281.2724	280.2646	$[M-H]^-$
Acrylamide	71.0366	72.0444	$[M+H]^+$
Ammonium bicarbonate	79.0269	60.9931	$[M-H]^-$
Aniline	93.0573	94.0651	$[M+H]^+$
Benzenesulphonate (acid form)	158.0038	156.9965	$[M-H]^-$
Benzopyrene	252.0939	253.1012	$[M+H]^+$
Biphenyl	154.0777	155.0855	$[M+H]^+$
Brillant blue	792.1222	769.1330	$[M-H]^-$
Butanedioic acid [(3.5-dimethoxyphenyl)methylene]-1-methyl ester	280.0941	281.1020	$[M+H]^+$
Butanol	74.0726	75.0804	$[M+H]^+$
Butyl alcohol	74.0726	75.0804	$[M+H]^+$
Butyl linoleate	336.3023	337.3101	$[M+H]^+$
Butylglycol	118.0988	119.1067	$[M+H]^+$
Castor oil diethanolamide	385.3187	386.3265	$[M+H]^+$
Ceteth-10	682.5226	683.5304	$[M+H]^+$
Ceteth-2	330.31285	331.32067	$[M+H]^+$
Ceteth-3	374.3391	375.3469	$[M+H]^+$
Ceteth-4	418.3653	419.3731	$[M+H]^+$
Ceteth-5	462.3915	463.3993	$[M+H]^+$
Ceteth-6	506.4177	507.4255	$[M+H]^+$
Ceteth-7	550.4439	551.4518	$[M+H]^+$
Ceteth-8	594.4701	595.4780	$[M+H]^+$
Ceteth-9	638.4963	639.5042	$[M+H]^+$
Citric acid	192.0276	191.0197	$[M-H]^-$
Cocamide MIPA	75.0679	76.0757	$[M+H]^+$
Cocamide monoethanolamide	243.2193	244.2271	$[M+H]^+$
Cocamidepropyl betaine	342.2877	343.2955	$[M+H]^+$
Cyclohexanol	100.0883	101.0961	$[M+H]^+$
Cyclohexanone	98.0726	99.0804	$[M+H]^+$
Cyclohexanone	98.0726	99.0804	$[M+H]^+$
D-Gluconic acid	196.0589	195.0510	$[M-H]^-$
D-Xylonic acid	166.0483	165.0405	$[M-H]^-$

Decyl octyl glucoside	320.2193	321.2272	[M+H]+
Dibutyl phthalate	278.1513	279.1591	[M+H]+
Diisobutyl phthalate	278.1513	279.1591	[M+H]+
Diisopropylnaphthalenesulphonic acid	292.1133	291.10604	[M-H]-
Dimethyl sulphoxide	78.0134	79.02121	[M+H]+
Dimethyl sulphoxide (DMSO)	78.0134	79.0212	[M+H]+
Dipropylene glycol methyl ether	148.1094	149.1172	[M+H]+
Dipropylene glycol methyl ether	148.1094	149.1172	[M+H]+
DMSO	78.0134	79.02121	[M+H]+
Dodecyl 4-hydroxybenzoate	306.2190	307.2268	[M+H]+
dodecyl sulphate	266.1552	267.1625	[M+H]+
Ethanol 2-(4-nonylphenoxy)	264.2084	265.2162	[M+H]+
Ethanol 2-[2-(4-octylphenoxy)ethoxy]-	294.2190	295.2268	[M+H]+
Ethanol 2-[2-[2-[2-(4-octylphenoxy)ethoxy]ethoxy]ethoxy]-	382.2714	383.2792	[M+H]+
Ethanol 2-[2-[2-[2-[4-(1,1,3,3-tetramethylbutyl)phenoxy]ethoxy]ethoxy]ethoxy]-	382.2714	383.2792	[M+H]+
Ethanol 2-[2-[2-[4-(1,1,3,3-tetramethylbutyl)phenoxy]ethoxy]ethoxy]-	338.2452	339.2530	[M+H]+
Ethanol 2-[2-[4-(1,1,3,3-tetramethylbutyl)phenoxy]ethoxy]-	294.2190	295.2268	[M+H]+
Ethanol 2-[4-(1,1,3,3-tetramethylbutyl)phenoxy]-	250.1927	251.2006	[M+H]+
Ethyl glycol	90.0675	91.0754	[M+H]+
Ethyl linoleate	306.2553	307.2632	[M+H]+
Ethylene glycol	62.0362	63.0441	[M+H]+
Ethylene glycol distearate	594.5582	595.5660	[M+H]+
Ethylene oxide	44.0262	45.0335	[M+H]+
Formaldehyde	30.0100	31.0178	[M+H]+
Formic acid	46.0055	44.9982	[M-H]-
Furfuryl alcohol	98.0362	99.0441	[M+H]+
Glutaric Anhydride	114.0312	115.0390	[M+H]+
Glycerol	92.0468	93.0546	[M+H]+
Glyceryl monolaurate	274.2139	275.2217	[M+H]+
Glyceryl monostearate	358.3078	359.3156	[M+H]+
Hexaethylene glycol	282.1684	281.1606	[M-H]-
Hexaethylene glycol monodecyl ether	422.3238	423.3316	[M+H]+
Hexaethylene glycol monotetradecyl ether	478.3864	479.3942	[M+H]+
Hexylene glycol	118.0988	119.1067	[M+H]+
Isobutyl alcohol	74.0726	75.0804	[M+H]+
Isononylphenol	220.1822	221.1900	[M+H]+

Isooctylphenol	206.1665	207.1743	$[M+H]^+$
Isophorone	138.1039	139.1117	$[M+H]^+$
Lauramide DEA	287.2455	288.2533	$[M+H]^+$
Lauramide DEA	288.2533	287.2455	$[M+H]^+$
laureth-2 sulfate (acid form)	354.2076	353.2003	$[M-H]^-$
Lauryldiemthylamine oxide	229.2400	230.2478	$[M+H]^+$
Lignosulphonates (Na. Ca. NH4) (acid form)	490.0967	489.0895	$[M-H]^-$
Mesityl oxide	98.0726	99.0804	$[M+H]^+$
Mesityl phenyl ketone	224.1196	225.1274	$[M+H]^+$
Methyl decanoate	186.1614	187.1693	$[M+H]^+$
Methyl octanoate	158.1301	159.1380	$[M+H]^+$
Methylmetacrylate	100.0519	101.0597	$[M+H]^+$
Methylparaben	152.0468	153.0546	$[M+H]^+$
Metilox	292.2033	293.2111	$[M+H]^+$
Monomethoxy PEG-11	516.3140	517.3219	$[M+H]^+$
Myreth-3	346.3078	347.3156	$[M+H]^+$
N, N- dimethyldecanamide	199.1931	200.2009	$[M+H]^+$
N,N-Diethyloctanamide	199.1931	200.2009	$[M+H]^+$
N,N-Dimethylformamide	73.0522	74.0600	$[M+H]^+$
N-Methyl-2-pyrrolidone	99.0679	100.0757	$[M+H]^+$
N-methylformamide	59.0366	60.0444	$[M+H]^+$
Nitrobenzene	123.0315	124.0393	$[M+H]^+$
NN-Dimethylformamide	73.0522	74.0600	$[M+H]^+$
Nonaethylene glycol monododecyl ether	582.4338	583.4416	$[M+H]^+$
Nonyl aldehyde	142.1352	143.1430	$[M+H]^+$
Nonylphenol. ethoxylated	264.2084	265.2162	$[M+H]^+$
o-(1-Ethylhexyl)phenol	206.1665	207.1743	$[M+H]^+$
o-(1-Propylpentyl)phenol	206.1665	207.1743	$[M+H]^+$
o-(1.1.3.3-Tetramethylbutyl)phenol	206.1665	207.1743	$[M+H]^+$
o-(1Methylheptyl)phenol	206.1665	207.1743	$[M+H]^+$
o-sec-Octylphenol	206.1665	207.1743	$[M+H]^+$
Octanol	130.1352	131.1430	$[M+H]^+$
Octylphenol	206.1665	207.1743	$[M+H]^+$
Oleth-3	400.3547	401.3625	$[M+H]^+$
Oleth-4	400.3547	401.3625	$[M+H]^+$
Oleyl alcohol	268.2761	269.2839	$[M+H]^+$
p-(1-Ethylhexyl)phenol	206.1665	207.1743	$[M+H]^+$
p-(1-Methylheptyl)phenol	206.1665	207.1743	$[M+H]^+$
p-(1-Methyloctyl)phenol	220.1822	221.1900	$[M+H]^+$

p-(1-Propylpentyl)phenol	206.1665	207.1743	$[M+H]^+$
p-Isooctylphenol	206.1665	207.1743	$[M+H]^+$
p-sec-Octylphenol	206.1665	207.1743	$[M+H]^+$
Pectin	194.0426	195.0499	$[M+H]^+$
PEG-4 sorbitan stearate	606.4338	607.4416	$[M+H]^+$
PEG-9	414.2460	415.2538	$[M+H]^+$
PEG11	502.2984	503.3062	$[M+H]^+$
PEG13	546.3246	547.3324	$[M+H]^+$
PEG14	634.3770	635.3849	$[M+H]^+$
Perfluorobutane sulfonate (acid form)	299.9503	298.9430	$[M-H]^-$
Perfluorononanoate (acid form)	463.9705	462.9632	$[M-H]^-$
Perfluorooctanesulfonate (acid form)	499.9375	498.9302	$[M-H]^-$
Perfluorooctanoate (acid form)	413.9737	412.9664	$[M-H]^-$
Phenol	94.0413	95.0491	$[M+H]^+$
Phenol 2-(1,1-dimethylhexyl)-	206.1665	207.1743	$[M+H]^+$
Phenol 2-(2-ethylhexyl)-	206.1665	207.1743	$[M+H]^+$
Phenol 2-(2-methylheptyl)-	206.1665	207.1743	$[M+H]^+$
Phenol 2-(5,5-dimethylhexyl)-	206.1665	207.1743	$[M+H]^+$
Phenol 3-(1,1-dimethylhexyl)-	206.1665	207.1743	$[M+H]^+$
Phenol 3-octyl-	206.1665	207.1743	$[M+H]^+$
Phenol 4-(1,1-dimethylhexyl)-	206.1665	207.1743	$[M+H]^+$
Phenol 4-(1,4-dimethylhexyl)-	206.1665	207.1743	$[M+H]^+$
Phenol 4-(2-ethylhexyl)-	206.1665	207.1743	$[M+H]^+$
Phenol 4-(2-methylheptyl)-	206.1665	207.1743	$[M+H]^+$
Phenol 4-(2-propylpentyl)-	206.1665	207.1743	$[M+H]^+$
Phenol 4-(5-methylheptyl)-	206.1665	207.1743	$[M+H]^+$
Phenol 4-(5,5-dimethylhexyl)-	206.1665	207.1743	$[M+H]^+$
Phenoxyethanol	138.0675	139.0754	$[M+H]^+$
Polyvinilalcohol	44.0262	43.0189	$[M-H]^-$
Potassium sorbate (acid form)	112.0524	111.0452	$[M-H]^-$
Propionic acid	74.0373	73.0295	$[M-H]^-$
Propylene glycol	76.05198	77.0597	$[M+H]^+$
propylene oxide	58.0419	57.0346	$[M-H]^-$
Propylparaben	180.0781	181.0859	$[M+H]^+$
Quinoline	129.0573	130.0651	$[M+H]^+$
Rapeseed oil	298.2866	299.2945	$[M+H]^+$
Silicate (acid from)	95.9879	94.9806	$[M-H]^-$
Sodium benzoate	122.0368	121.0295	$[M-H]^-$
Sodium benzoate	122.0368	123.0440	$[M+H]^+$

Sodium citrate	192.0270	189.0041	[M-H]-
Sodium decyl sulfate	239.1323	237.1166	[M-H]-
Sodium lignosulfonate	488.0816	487.0738	[M-H]-
Sodium xylenesulfonate	188.0507	187.0434	[M-H]-
Sorbitan monolaurate	346.23499	347.24282	[M+H]+
Sorbitan monostearate	430.3289	431.3367	[M+H]+
Sorbitan oleate	428.3138	429.3211	[M+H]+
Sorbitan tristearate	962.8508	963.8587	[M+H]+
Steareth-2	358.34415	359.35197	[M+H]+
Steareth-4	446.3966	447.4044	[M+H]+
Steareth-6	534.4490	535.4568	[M+H]+
Steareth-7	578.4752	579.4831	[M+H]+
Stearyldiethanolamine	357.3601	358.3680	[M+H]+
TEA (Triethylamine)	101.1199	102.1277	[M+H]+
Tetrahydrofurfuryl Alcohol	102.0675	103.0754	[M+H]+
Tetrasodium EDTA	288.0599	287.0521	[M-H]-
Trideceth-3	332.2921	333.3000	[M+H]+
Triethanolamine	149.1046	150.1125	[M+H]+
Triethylene glycol monometyl ether	164.1043	165.1121	[M+H]+
Tristyrylphenol	400.1827	399.1754	[M-H]-
Urea	60.0318	61.0396	[M+H]+
Urea-formaldehyde	90.0423	91.0502	[M+H]+
Vinylacetate	86.0368	87.0441	[M+H]+
Xylene	318.2342	319.2420	[M+H]+
Sorbitan monooleate	428.3138	427.3059	[M-H]-
di-2-ethylhexyl sulfosuccinic acid	422.2333	421.2265	[M-H]-
Maltodextrin	342.1162	341.1093	[M-H]-
Lecithin	757.5621	802.5586	[M-H]-
Ascorbic acid	176.0321	175.0248	[M-H]-
Potassium (E.E)-hexa-2.4-dienoate	150.0083	175.0248	[M+H]+
Sucrose (alpha-D-Glucopyranoside. beta-D-Fructofuranosyl)	342.1162	341.1089	[M-H]-
Trimethylolpropane	134.0943	135.1016	[M+H]+
2,2'-iminodi(ethylamine) (N-2-aminoethyl-1,2-ethanediamine)	103.1109	104.1182	[M+H]+
Dextrose (glucose)	180.0634	179.0563	[M-H]-
4-nitrophenol	139.0269	138.0197	[M-H]-
Hexamethylene tetramine	140.1062	141.1135	[M+H]+
Isopropyl myristate	270.2559	271.2744	[M+H]+
Hexa-2,4-dienoic acid (sorbic acid)	112.0524	113.0597	[M+H]+
Hexa-2,4-dienoic acid (sorbic acid)	112.0524	111.0452	[M-H]-

Sodium L-glutamate	147.05261	148.0604	$[M+H]^+$
2-(3-tert-butyl-2-hydroxy-5-methylphenyl)-5-chloro-2H-benzotriazole	315.1138	314.1066	$[M+H]^+$
Edetic acid (EDTA) -Ethylenediaminetetraacetic acid	292.0907	293.0979	$[M+H]^+$
Propylparaben (propyl 4-hydroxybenzoate)	180.0786	179.0714	$[M-H]^-$
diethyl malonate	160.0736	161.0808	$[M+H]^+$
Bis(2,2,6,6-tetramethyl-4-piperidyl) sebacate	480.3927	481.4000	$[M+H]^+$
D-Glucose. hydrate	180.1559	198.0972	$[M+H]^+$

Table S4. Tentative identification of co-formulants by Acclaim Surfactant Plus column.

Compound	Molecular formula	Retention time (min)	Adduct	Characteristic ions	
				Theorical mass	Mass error
Octyl 4-methylbenzenesulfonate	$C_{15}H_{24}O_3S$	7.89	$[M-H]^-$	283.1371	3.420
2-[2-(4-octylphenoxy)ethoxy]-ethanol	$C_{20}H_{34}O_4$	1.33	$[M+H]^+$	339.2530	-8.448
N,N-dimethyldecanamide	$C_{12}H_{25}NO$	9.84	$[M+H]^+$	200.2009	-3.102
Citric acid	$C_6H_8O_7$	0.67	$[M-H]^-$	191.0197	1.837
Dibutyl phthalate	$C_{16}H_{22}O_4$	9.71	$[M+H]^+$	279.1591	-3.818
Lauramide DEA	$C_{16}H_{33}NO_3$	9.41	$[M+H]^+$	288.2533	-4.303
Diethylene glycol n-butyl ether	$C_8H_{18}O_3$	1.62	$[M+H]^+$	163.1325	-3.561

Table S5. Identification of co-formulants by suspect analyses in PPPs. Compounds in bold were confirmed with their analytical standard.[a]

Nº	Compound name	Molecular formula	Retention time	Adduct	Characteristic ions		Fragment ions			PPP	LvC
					Theoretical mass	Mass error (ppm)	Theoretical mass	Molecular formula	Mass error (ppm)		
**1**	**4-Dodecylbenzenesulfonic acid**	$C_{18}H_{30}O_3S$	1.24	$[M-H]^-$	325.1843	-0.640	79.9574<br>183.0121	SO_3<br>$C_8H_7O_3S$	-2.578<br>-0.390	P2, P3, P6, P7, P11-P14, P17, P19, P20	1
**2**	4-Undecylbenzenesulfonic acid	$C_{17}H_{28}O_3S$	1.24	$[M-H]^-$	311.1686	-0.490	79.9574<br>183.0121	SO_3<br>$C_8H_7O_3S$	-3.578<br>0.047	P2, P3, P6, P7, P11- P14, P17, P19, P20	2
**3**	4-Decylbenzenesulfonic acid	$C_{16}H_{26}O_3S$	1.24	$[M-H]^-$	297.1530	-1.045	79.9574<br>183.0121	SO_3<br>$C_8H_7O_3S$	-2.578<br>0.102	P2, P3, P6, P7, P11-P14, P16-P20	2
**4**	9-Octadecenamide	$C_{18}H_{35}NO$	2.18	$[M-H]^-$	280.2646	-0.554	97.1012<br>247.2420	C_7H_{13}<br>$C_{18}H_{31}$	3.121<br>-2.134	All except for P15, P16, P17, P18	2
**5**	1-Methylpyrrolidin-2-one/N-Methyl-2-pyrrolidone	C_5H_9NO	2.25	$[M+H]^+$	100.0757	1.689	98.0600	C_5H_8NO	1.933	P4, P5	2
**6**	4-Nonyl benzenesulfonic acid	$C_{15}H_{24}O_3S$	1.29	$[M-H]^-$	283.1373	-0.793	79.9574<br>183.0121	SO_3<br>$C_8H_7O_3S$	-5.579<br>0.867	P2, P3, P6, P7, P12-P17, P19, P20	2
**7**	**4-Sec-butyl-2,6-di-tert-butylphenol**	$C_{18}H_{30}O$	1.91	$[M+H]^+$	263.2369	-3.252	245.2264<br>207.1742	$C_{18}H_{29}$<br>$C_{14}H_{23}O$	-0.497<br>-0.182	P7, P12, P15, P19	1
				$[M-H]^-$	261.2238	0.818					

**8**	2-(4-Methyl-1-piperazinyl)ethanol	$C_7H_{16}N_2O$	6.01	$[M+H]^+$	145.1335	-1.333	88.0757<br>98.0600	$C_4H_{10}NO$<br>C_5H_8NO	3.060<br>1.219	P4, P5	2
**9**	**N, N-dimethyldecanamide/N,N-Diethyloctanamide**	$C_{12}H_{25}NO$	1.92	$[M+H]^+$	200.2009	-3.089	102.0913<br>116.1070<br>130.1264<br>198.1852	$C_5H_{12}ON$<br>$C_6H_{14}ON$<br>$C_7H_{16}ON$<br>$C_{12}H_{24}ON$	0.269<br>0.049<br>-0.111<br>-0.351	P4, P5, P6, P7, P16, P17, P20	1
**10**	**Lauramide DEA (N,N-Bis(2-hydroxyethyl)dodecanamide)**	$C_{16}H_{33}NO_3$	2.54	$[M+H]^+$	288.2533	-3.048	70.0651<br>88.0766<br>106.0863	C_4H_8N<br>$C_4H_{10}NO$<br>$C_4H_{12}O_2N$	8.195<br>4.536<br>1.177<br>-2.318	P1, P2, P5, P6, P7, P8-P11, P13, P17, P20	1
**11**	Metilox	$C_{18}H_{28}O_3$	1.82	$[M+H]^+$	293.2111	-2.704	291.1955<br>275.2006	$C_{10}H_{31}O_{10}$<br>$C_{17}H_{23}O_3$	-3.459<br>-2.621	P3, P5, P7, P8	2
**12**	2-(p-Octylphenoxy)ethanol	$C_{16}H_{26}O_2$	1.91	$[M+H]^+$	251.2006	-3.699	81.0699	C_6H_9	5.219	P1, P3, P5, P6, P7	2
**13**	Tetraethytlene glycol monohexadecyl ether (Ceteth-4)	$C_{24}H_{50}O_5$	2.20	$[M+H]^+$	419.3731	-3.620	89.0597<br>133.0859<br>177.1121	$C_4H_9O_2$<br>$C_6H_{13}O_3$<br>$C_8H_{17}O_4$	4.760<br>-0.457<br>0.759	P3, P6, P7	2
**14**	2-[2-[4-(1,1,3,3-tetramethylbutyl)phenoxy]ethoxy]-ethanol	$C_{18}H_{30}O_3$	1.91	$[M+H]^+$	295.2268	-1.943				P1-P3, P5-P7, P13, P14	3
**15**	Triethylene glycohexamonohexadecyl ether (Ceteth-3)	$C_{22}H_{46}O_4$	2.24	$[M+H]^+$	375.3469	-2.756	89.0597	$C_4H_9O_2$	4.423	P3, P6, P7, P8, P12, P14	2
**16**	Lauryldiemthylamine oxide	$C_{14}H_{31}NO$	16.69	$[M+H]^+$	230.2478	-2.084	133.1010<br>60.0443	$C_{10}H_3$<br>C_2H_6ON	-2.156<br>5.986	P1, P8, P10	2
**17**	Triethyleneglycol monooleyl ether (Oleth-3)	$C_{24}H_{48}O_4$	2.24	$[M+H]^+$	401.3625	-1.727				P3, P6, P7, P12	4
**18**	Laureth-2 sulfate	$C_{16}H_{34}O_6S$	13.16	$[M-H]^-$	353.2003	-0.554	79.9574<br>97.0659	SO_3<br>C_6H_9O	-1.870	P3, P11, P18, P19	2
**19**	C16E5 (Hexadecyl pentaethylene glycol ether) (Ceteth-5)	$C_{26}H_{54}O_6$	2.24	$[M+H]^+$<br>$[M+NH_4]^+$	463.3993<br>480.4245	-2.300<br>-3.046	89.0597<br>133.0859<br>177.1121	$C_4H_9O_2$<br>$C_6H_{13}O_3$<br>$C_8H_{17}O_4$	5.546<br>-0.908<br>-1.725	P3, P6, P7	2

**20**	Ethyl linoleate	$C_{20}H_{34}O_2$	1.65	$[M+H]^+$	307.2632	-3.102				P2, P6, P12, P13, P19, P20	4
**21**	3-hexenal/mesityl oxide	$C_6H_{10}O$	1.91	$[M+H]^+$	99.0804	1.815				P2	4
**22**	2-Phenylpropanal	$C_9H_{10}O$	1.65	$[M+H]^+$	135.0804	-3.131				P1, P2, P4-P10, P16, P20	4
**23**	Xylene	$C_{24}H_{30}$	1.87	$[M+H]^+$	319.2420	2.883				P1- P4, P6, P15	4
**24**	Dodecyl 4-hydroxybenzoate	$C_{19}H_{30}O_3$	1.87	$[M+H]^+$	307.2268	-2.960	305.2111	$C_{19}H_{29}O_3$	-0.211	P1	2
**25**	Dibutyl phthalate	$C_{16}H_{22}O_4$	1.78	$[M+H]^+$	279.1591	-2.740	149.0233<br>177.0910<br>217.1223<br>277.1434	$C_8H_5O_3$<br>$C_{11}H_{13}O_2$<br>$C_{14}H_{17}O_2$<br>$C_{16}H_{21}O_4$	-1.480<br>-5.790<br>8.382<br>-1.480	All except for P13, P14, P15, P17, P18	2
**26**	Phenoxyethanol	$C_8H_{10}O_2$	1.65	$[M+H]^+$	139.0754	-3.159				P1, P3, P5, P6, P7	4
**27**	**1-Ethylpyrrolidin-2-one**	$C_6H_{11}NO$	2.54	$[M+H]^+$	114.0913	-0.142	112.0757	$C_6H_{10}NO$	0.799	P1, P4, P13, P14, P17-P20	1
**28**	2-(4-Nonylphenoxyethanol)	$C_{17}H_{28}O_2$	1.82	$[M+H]^+$	265.2162	-2.505				P5, P7	4
**29**	**Sorbitan monostearate**	$C_{24}H_{46}O_6$	1.51	$[M-H]^-$	429.3222	-2.156	59.0126<br>279.2330	$C_2H_3O_2$<br>$C_{18}H_{31}O_2$	-3.319<br>3.127	P2, P6, P7, P8, P12, P13, P19	1
**30**	Castor oil diethanolamide	$C_{22}H_{43}NO_4$	2.16	$[M+H]^+$	386.3265	-3.351				P2, P6, P7	4
**31**	1-Methoxy-2-propanol/ 2-ethoxyethanol/2-Methoxypropanol/Ethyl glycol	$C_4H_{10}O_2$	1.86	$[M+H]^+$	91.0754	1.961				P2	4
**32**	Cocamidepropyl betaine	$C_{19}H_{38}N_2O_3$	14.57	$[M+H]^+$	343.2955	-2.542	240.2315<br>183.1738<br>95.0860<br>109.1015	$C_{15}H_{30}NO$<br>$C_{12}H_{23}O$<br>C_7H_{10}<br>C_8H_3	-2.169<br>-2.740<br>2.030<br>0.486	P1, P6, P7, P20	2
**33**	2,2-Dimethylocta-3,4-dienal	$C_{10}H_{16}O$	1.82	$[M+H]^+$	153.1274	-3.052	133.0859	$C_6H_{13}O_3$	-0.231	P5, P6, P7	2

							69.0699	C_5H_9	7.140		
**34**	Tetraethylene glycol monooctadecyl ether (Steareth-4)	$C_{26}H_{54}O_5$	19.43	$[M+H]^+$	447.4044	-2.140				P1, P3, P4, P6, P7, P12, P14,	
**35**	4-Phenylcyclohexanone	$C_{12}H_{14}O$	1.86	$[M+H]^+$	175.1117	-2.701				P7	4
**36**	**Aniline**	C_6H_7N	2.29	$[M+H]^+$	94.0651	2.294				P1, P9-P11, P15, P19, P20	1
**37**	Diethytlene glycol monohexadecyl ether (Ceteth-2)	$C_{20}H_{42}O_3$	20.5	$[M+H]^+$	331.3207	-2.184				P6, P7, P8, P12	3
**38**	Triethylene glycol monotetradecyl ether (Myreth-3)	$C_{20}H_{42}O_4$	19.04	$[M+H]^+$	347.3156	-2.307	89.0597<br>133.0859	$C_4H_9O_2$<br>$C_6H_{13}O_3$	4.311<br>-1.113	P1-P4, P6, P7, P9, P12, P15	2
**39**	Trideceth-3	$C_{19}H_{40}O_4$	18.14	$[M+H]^+$	333.2999	-3.428	89.0597<br>70.0413	$C_4H_9O_2$<br>C_4H_6O	4.199<br>-3.656	P8, P9, P18	2
**40**	Cocamide monoethanolamide	$C_{14}H_{29}NO_2$	16.18	$[M+H]^+$	244.2271	-2.179				P5, P6, P14	4
**41**	Hexaethylene glycol monohexadecyl ether (Ceteth-6)	$C_{28}H_{58}O_7$	2.24	$[M+H]^+$<br>$[M+NH_4]^+$	507.4255<br>524.4508	-2.993<br>-2.487	89.0597<br>133.0859<br>177.1121	$C_4H_9O_2$<br>$C_6H_{13}O_3$<br>$C_8H_{17}O_4$	3.749<br>-1.734<br>-1.725	P6, P7, P12, P14	2
**42**	Ethanol 2-[2-[2-[2-(4-octylphenoxy)ethoxy]ethoxy]ethoxy]/Ethanol 2-[2-[2-[2-[4-(1.1.3.3-tetramethylbutyl)phenoxy]ethoxy]ethoxy]ethoxy]-	$C_{22}H_{38}O_5$	1.78	$[M+H]^+$	383.2792	-2.375				P1, P2, P3, P4, P8, P9, P10, P20	4
**43**	2-Methylisothiazolone	C_4H_5NOS	2.55	$[M+H]^+$	116.0165	-0.621				P1, P8, P9	4
**44**	Decaethylene glycol monohexadecyl ether (Ceteth-10)	$C_{36}H_{74}O_{11}$	23.48	$[M+H]^+$	683.5304	-3.832				P6	3
**45**	2,2-Dimethylocta-3.4-dienal	$C_{10}H_{16}O$	1.82	$[M+H]^+$	153.1274	-2.554				P4, P5, P6	4

**46**	Heptaethylene glycol monohexadecyl ether (Ceteth-7)	$C_{30}H_{62}O_8$	2.33	$[M+H]^+$	551.4518	-0.495				P6, P12	3
**47**	**1-Naphthalenesulfonic acid**	$C_{10}H_8O_3S$	1.23	$[M-H]^-$	207.0121	-4.025	79.9574<br>143.0502	O_3S<br>$C_{10}H_7O$	-3.453<br>-1.688	All except for P2, P6, P7, P12 and P17	1
**48**	2-[2-[2-(4-Octylphenoxy)ethoxy]ethoxy]ethanol	$C_{20}H_{34}O_4$	1.86	$[M+H]^+$	339.2530	-1.707				P8, P9	4
**49**	3,6,9,12-tetraoxapentacosan-1-ol	$C_{21}H_{44}O_5$	1.86	$[M+H]^+$	377.3262	-3.501				P8, P9, P18	4
**50**	4-Phenylsulfonic acid	$C_6H_6O_4S$	1.23	$[M-H]^-$	172.9914	-4.886	93.0346<br>79.9574	C_6H_5O<br>SO_3	-1.734<br>-3.578	P8	2
**51**	Hexapropylene glycol monobutyl ether	$C_{22}H_{46}O_7$	1.86	$[M+H]^+$	423.3316	-4.243				P8	4
**52**	Sodium decyl sulfate	$C_{10}H_{22}O_4S$	1.23	$[M-H]^-$	237.1166	-1.383	79.9574	SO_3	2.320	P8	2
**53**	**Triethylene glycol monometyl ether**	$C_7H_{16}O_4$	1.81	$[M+H]^+$	165.1121	4.694	103.0754	$C_5H_{11}O_2$	3.433	P2, P6, P10, P11, P13, P14, P16-P18	1
**54**	**2-Amino-1,3-dimethylbenzene (2,6-Dimethylaniline)**	$C_8H_{11}N$	2.28	$[M+H]^+$	122.0964	-1.079	105.0699<br>107.0731	C_8H_9<br>$C_6H_7N_2$	-0.704<br>2.219	P9, P11, P15, P19	1
**55**	Butyl-1-naphthalenesulfonic acid	$C_{14}H_{16}O_3S$	1.20	$[M-H]^-$	263.0747	-0.241	79.9574	SO_3	-5.205	P10, P14, P15, P16	2
**56**	2,6-Di-tert-butylnaphthalene-1-sulfonic acid	$C_{18}H_{24}O_3S$	1.24	$[M-H]^-$	319.1373	-0.608	79.9574	SO_3	-5.704	P10, P15	2
**57**	8-(2-Methyl-2-undecanyl)-1-naphthalenesulfonic acid	$C_{22}H_{32}O_3S$	1.24	$[M-H]^-$	375.1999	-0.865	79.9574	SO_3	-3.738	P10	2
**58**	Butanedioic acid [(3.5-dimethoxyphenyl) methylene]-1-methyl ester	$C_{14}H_{16}O_6$	1.78	$[M+H]^+$	281.1020	-3.368				P10	4

**59**	D-Gluconic acid	$C_6H_{12}O_7$	1.67	$[M-H]^-$	195.0510	-3.231	179.0561<br>165.0405	$C_6H_{11}O_6$<br>$C_5H_9O_6$	1.427<br>0.761	P10	2
**60**	D-Xylonic acid	$C_5H_{10}O_6$	1.76	$[M-H]^-$	165.0405	-4.769	147.0299<br>129.0193	$C_5H_7O_5$<br>C_4H_2O	1.634<br>-0.737	P10	2
**61**	Glutaric Anhydride	$C_5H_6O_3$	2.55	$[M+H]^+$	115.0390	-0.920	85.0284<br>73.0284	$C_4H_5O_2$<br>$C_3H_5O_2$	4.870<br>7.587	P10	2
**62**	Sodium xylenesulfonate	$C_8H_9O_3SH$	1.17	$[M-H]^-$	185.0278	-4.061	79.9574<br>121.0659	O_3S<br>C_8H_9O	-3.453<br>-0.012	P11, P14, P18	2
**63**	Undecaethylene glycol (PEG11)	$C_{22}H_{46}O_{12}$	1.85	$[M+H]^+$	503.3062	-4.097				P12	4
**64**	2-(2-Octadecoxyethoxy)ethanol) (Steareth-2)	$C_{22}H_{46}O_3$	21.8	$[M+H]^+$	359.3520	-2.748				P12, P14	4
**65**	Butyl linoleate	$C_{22}H_{40}O_2$	1.98	$[M+H]^+$	337.3101	-2.957				P13	4
**66**	Citric acid	$C_6H_8O_7$	2.52	$[M-H]^-$	191.0197	-4.236	129.0193<br>111.0088<br>87.0088	$C_5H_5O_4$<br>$C_5H_3O_3$<br>$C_3H_3O_3$	-0.582<br>-1.179<br>-2.419	P6, P7, P15	2
**67**	Diisopropylnaphthalenesulphonic acid	$C_{16}H_{20}O_3S$	1.19	$[M-H]^-$	291.1060	-1.019	79.9574	O_3S	-0.356	P15	2
**68**	Triethanolamine	$C_6H_{15}NO_3$	5.99	$[M+H]^+$	150.1125	-2.016	130.0863<br>132.1019	$C_6H_{12}NO_2$<br>$C_6H_{14}NO_2$	-1.116<br>-1.326	P16, P17, P18, P19, P20	2
**69**	Quinoline	C_9H_7N	2.28	$[M+H]^+$	130.0651	-1.450				P16	4
**70**	C16E8 (Octaethylene glycol monohexadecyl ether (Ceteth-8))	$C_{32}H_{66}O_9$	2.28	$[M+H]^+$<br>$[M+NH_4]^+$	595.47796<br>612.5028	-2.458<br>-2.896	89.0597<br>133.0859<br>177.1121	$C_4H_9O_2$<br>$C_6H_{13}O_3$<br>$C_8H_{17}O_4$	4.086<br>-1.734<br>-2.346	P3, P6, P7	2

[a] Abbreviation: LvC: Level of confidence; P1: Voliam Targo; P2: Kabuto JED; P3: Mavita; P4: Cidely Top; P5: Dynali; P6: Lexor; P7: Score 25; P8: Dagonis; P9: Coragen 20 SC; P10: Altacor; P11: Ampligo; P12: Nomada; P13: Duaxo; P14: Ortiva Top, P15: Flint max; P16: Topas; P17: Massocur 12.5; P18: Impact Evo star; P19: Latino (Mitrus); P20: Impala star.

Table S6. Properties of co-formulants confirmed in PPPs

Co-formulant	Properties	Reference
Dodecylbenzenesulfonic acid	Good dispersing, emulsifying, wetting, and foaming properties	25,26
1-Naphthalene sulfonic acid		
Triethylene glycol monomethyl ether	Solvent in PPPs due to its high solvency glycol ether with excellent coupling properties	27
N,N-dimethyldecanamide	Solvent for active ingredients in agricultural formulations	13
4-Sec-butyl-2,6-di-tert-butylphenol	Preservative in non-toxic aqueous pesticides	28
Lauramide DEA	Thickening, foam enhancer, and stabilizer in cosmetics and shampoos	2
1-Ethyl-2-pyrrolodine	Water solubility and solvent power and it is used for different applications including pesticides, pharmaceutical industry, and cosmetic products	29
Sorbitan monostearate (Span 60)	Emulsifier and stabilizer agent in medicine, cosmetics, food, pesticide, coatings, plastic and textiles industries	30
2,6-Dimethylaniline	Chemical intermediate in the manufacture of pesticides	31
Aniline	Use in agricultural fungicides and herbicides	2
Palmitamide	Nonionic surfactant derived from the palm oil	32
N-Lauryldiethanolamine	Antistatic agent and cosmetic ingredient that belongs to the class of ionizable surfactants	33

Table S7. Evaluation of co-formulant recoveries (spiked concentration: 50 µg/L) in Kabuto® JED.

Compound	Mean recovery (%)	RSD (n = 6)
Dodecylbenzenesulfonic acid	113	10 %
1-Naphthalene sulfonic acid	101	3 %
Triethylene glycol monomethyl ether	105	2 %
N,N-dimethyldecanamide	112	5 %
4-Sec-butyl-2,6-di-tert-butylphenol	88	2 %
Lauramide DEA	118	1 %
1-Ethyl-2-pyrrolidone	109	18 %
Sorbitan monostearate (Span 60)	99	19 %
2,6-dimethylaniline	89	1 %
Aniline	98	10 %
Palmitamide	115	9 %
N-Lauryldiethanolamine	116	9 %

Figure S1. Extracted ion chromatograms and spectra of octyl 4-methylbenzenesulfonate: (A) Lexor with Shodex column; (B) Lexor with Acclaim column (C) Full MS spectrum with Shodex column (D) Full MS spectrum with Acclaim (E) Theorical Full MS spectrum.

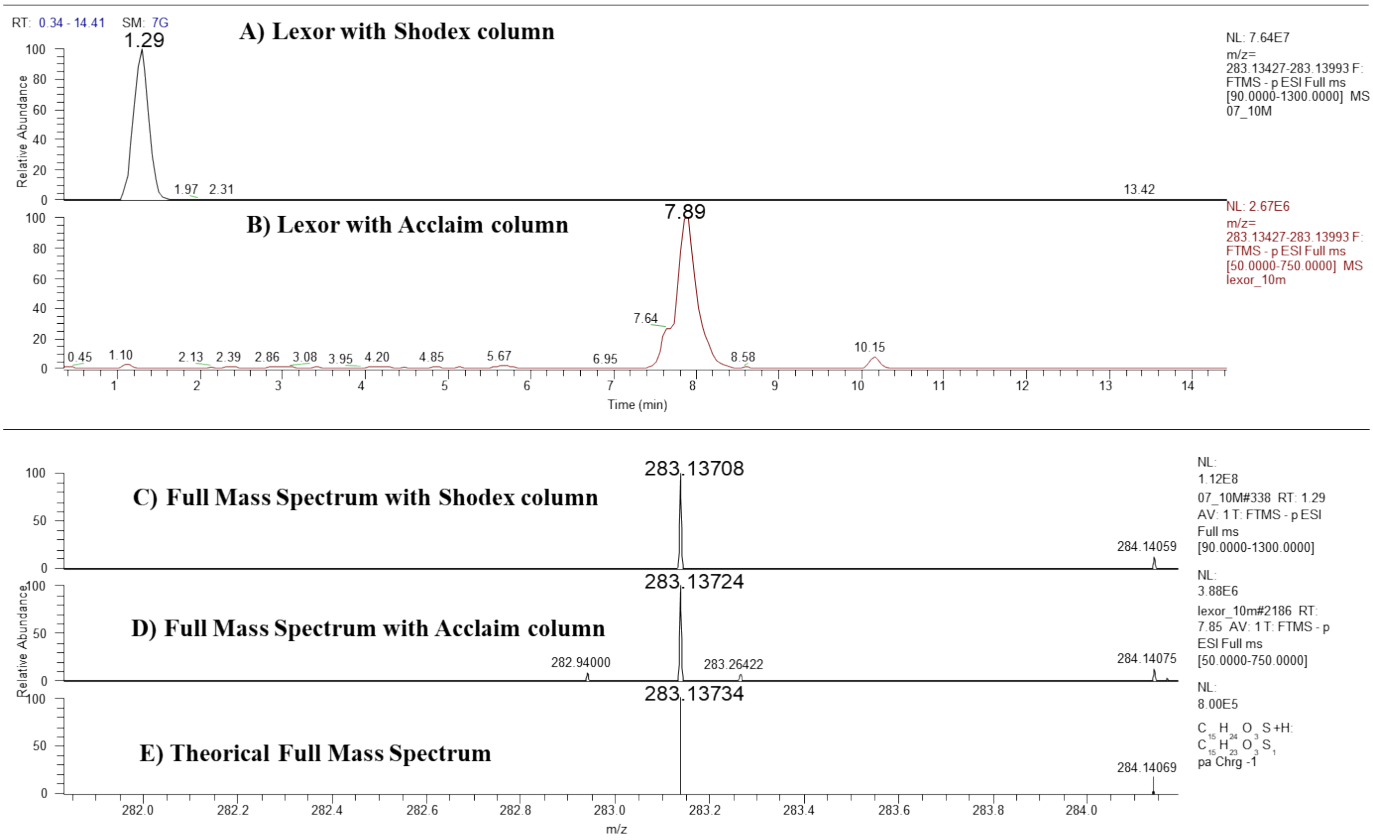

Supplement: Supplementary file 1 — jf3c03600_si_001.pdf [file jf3c03600_si_001.pdf]
